# Feature Selection Using Enhanced Particle Swarm Optimisation for Classification Models

**DOI:** 10.3390/s21051816

**Published:** 2021-03-05

**Authors:** Hailun Xie, Li Zhang, Chee Peng Lim, Yonghong Yu, Han Liu

**Affiliations:** 1 Computational Intelligence Research Group, Department of Computer and Information Sciences, Faculty of Engineering and Environment, University of Northumbria, Newcastle upon Tyne NE1 8ST, UK; hailun.xie@northumbria.ac.uk; 2 Institute for Intelligent Systems Research and Innovation, Deakin University, Waurn Ponds, VIC 3216, Australia; chee.lim@deakin.edu.au; 3 College of Tongda, Nanjing University of Posts and Telecommunications, Nanjing 210049, China; yuyh@njupt.edu.cn; 4 College of Computer Science and Software Engineering, Shenzhen University, Shenzhen 518060, China; han.liu@szu.edu.cn

**Keywords:** feature selection, evolutionary algorithm, particle swarm optimisation, classification

## Abstract

In this research, we propose two Particle Swarm Optimisation (PSO) variants to undertake feature selection tasks. The aim is to overcome two major shortcomings of the original PSO model, i.e., premature convergence and weak exploitation around the near optimal solutions. The first proposed PSO variant incorporates four key operations, including a modified PSO operation with rectified personal and global best signals, spiral search based local exploitation, Gaussian distribution-based swarm leader enhancement, and mirroring and mutation operations for worst solution improvement. The second proposed PSO model enhances the first one through four new strategies, i.e., an adaptive exemplar breeding mechanism incorporating multiple optimal signals, nonlinear function oriented search coefficients, exponential and scattering schemes for swarm leader, and worst solution enhancement, respectively. In comparison with a set of 15 classical and advanced search methods, the proposed models illustrate statistical superiority for discriminative feature selection for a total of 13 data sets.

## 1. Introduction

The knowledge discovery processes in real-world applications often involve datasets with large numbers of features [1]. The high dimensionalities of datasets increase the likelihood of overfitting and impair generalization capability. Besides that, the inclusion of redundant or even contradictory features can severely reduce the performance of classification, regression and clustering algorithms [2]. As a result, feature selection and dimensionality reduction become critical in overcoming the aforementioned challenges by eliminating certain irrelevant and redundant features while identifying the most effective and discriminative ones [3,4]. Moreover, for datasets with high dimensionalities, it is computationally impractical to conduct an exhaustive search of all possible combinations of the feature subsets [5]. In addition, the search landscape becomes extremely complicated, owing to the sophisticated confounding effects of various feature interactions in terms of redundancy and complementarity [6]. Therefore, effective and robust search methods are required to thoroughly explore the complex effects of feature interactions while satisfying the constraints of practicality in term of computational cost to undertake large-scale feature selection tasks.

Evolutionary Computation (EC) techniques have been widely employed to comprehensively explore the complex effects of feature interactions, owing to the significant capability of EC in finding global optimality [4]. Inspired by natural evolution, EC techniques employ a population-based evolving mechanism to supervise the individual solutions to move towards the promising search territory iteratively and identify the global optima. In EC-based feature selection methods, the coevolution mechanisms based on diverse evolving operators, e.g., crossover and mutation, are capable of producing various feature representations of the original problem in one single run. Therefore, the confounding effects of feature interactions can be thoroughly explored through the evaluation of various feature constitutions during the iterative process. The effectiveness and superiority of various EC techniques over other methods in undertaking feature selection tasks have been extensively verified in many existing studies, such as feature optimisation using Genetic Algorithm (GA) [7], Differential Evolution (DE) [8,9], Particle Swarm Optimisation (PSO) [10], Moth-flame optimisation (MFO) [11], Firefly Algorithm (FA) [3,12], Ant Colony Optimisation (ACO) [13], Grey Wolf Optimisation (GWO) [14], Whale Optimisation Algorithm (WOA) [15], and Sine Cosine Algorithm (SCA) [16]. Nevertheless, the empirical studies indicated that these original EC algorithms tend to be trapped in local optima, and they can be further improved in terms of search diversity and capability of avoiding local stagnation.

As one of the most acknowledged and widely-used EC algorithms, PSO has been adopted in various optimisation problems, owing to its simplicity, fast convergence speed, as well as effectiveness and robust generalization capability. In PSO, each particle adjusts its search trajectory by learning from two historical best experiences, i.e., its own best position and the global best solution. Despite the great advantages in following both the local and global best signals, PSO suffers from the local optima traps as well as inefficient fine-tuning capabilities owing to its working principles [17,18,19]. As an example, PSO lacks the operation of exchanging information between particles, owing to the fact that only the global best solution is exploited as the reference for coevolution [20]. Secondly, the swarm often tends to revisit previously explored regions, owing to the strict adherence to the historical best experiences of each particle [21]. These limitations in the original PSO model severely constrain the search diversity and search scope, hence resulting in early stagnation and premature convergence. Such constraints of the PSO algorithm become worse when undertaking feature selection tasks with complex problem landscapes.

In this research, we propose two enhanced PSO models to address the identified limitations of the original PSO algorithm as well as undertake complex feature selection problems. Specifically, the research overcomes the lack of cooperation between individual particles and ineffectiveness of search owing to frequent re-visits to previously explored regions in the original PSO model. The proposed PSO models employ several key strategies, including leader/exemplar generation using dynamic absorption of elicit genes, search operations with differentiated nonlinear trajectories, exploitation schemes for swarm leader enhancement, as well as re-dispatching mechanisms for enhancement of the worst solutions. These strategies work cooperatively as augmentations to accelerate convergence while preserving diversity. A summary of the research contributions is presented, as follows:

- Two new PSO variants for feature selection are proposed to overcome two major shortcomings of the original PSO algorithm, i.e., premature convergence and weak local exploitation capability around the near optimal solutions.
- The first proposed PSO model, i.e., PSOVA1 (PSO variant 1), comprises the following mechanisms: (1) a modified PSO operation with rectified global and personal best signals, (2) spiral search based local exploitation, (3) Gaussian distribution based swarm leader enhancement, as well as (4) mirroring and DE mutation operations for worst solution improvement.
- The second proposed PSO model, i.e., PSOVA2 (PSO variant 2), enhances PSOVA1 through four mechanisms: (1) an adaptive exemplar breeding mechanism incorporating multiple optimal signals, (2) search coefficient generation using sine, cosine, and hyperbolic tangent functions, (3) worst solution enhancement using a hybrid re-dispatching scheme, and (4) an exponential exploitation scheme for swarm leader improvement. Moreover, the search diversity and scopes in PSOVA2 are further elevated in comparison with those of PSOVA1. This is owing to the adoption of diverse exemplars to guide the search in each dimension, as well as the employment of versatile search trajectories to calibrate the particle positions.
- Evaluation using 13 datasets with a wide spectrum of dimensionalities: the empirical results indicate that both proposed models outperform five classical search methods and ten advanced PSO variants with significant advantages, evidenced by the statistical test outcomes.

The rest of the paper is organized as follows. Section 2 introduces the original and diverse PSO models, and their applications to feature selection. We present the two proposed PSO models with elaborations and analysis for each proposed enhancement in Section 3 and Section 4, respectively. Section 5 discusses the evaluation of the proposed and the baseline search methods on a variety of feature selection tasks. Conclusions are drawn and future research directions are presented in Section 6.

## 2. Related Studies

In this section, we firstly introduce the original PSO model. Then, the state-of-the-art PSO variants are presented. We also conduct a literature review on the application of PSO variants to feature selection. Finally, we discuss the motivation of this research.

### 2.1. Particle Swarm Optimisation

PSO is a population-based self-adaptive optimisation technique developed by Kennedy and Eberhart [22] based on swarm social behaviors, such as fish in a school and birds in a flock. The PSO algorithm conducts search in the landscape of the objective function by adjusting the trajectories of individual particles in a quasi-stochastic manner [23,24]. Each particle adjusts its velocity and position by following its own best experience in history and the global best solution of the swarm. In the PSO model, the updating equations of the velocity *v_id_^t+^*^1^ and position *x_id_^t+^*^1^ of the *i*th particle at the *d*th dimension are prescribed in Equations (1) and (2) [22]:

(1) $$v_{id}^{t+1}=w\times v_{id}^{t}+c_{1}\times r_{1}\times \left(pbest_{id}-x_{id}^{t}\right)+c_{2}\times r_{2}\times \left(gbest_{d}-x_{id}^{t}\right)$$

(2) $$x_{id}^{t+1}=x_{id}^{t}+v_{id}^{t+1}$$

where *v_i_* and *x_i_* represent the velocity and position of the *i*th particle, while *pbest_i_* an *gbest* represent the historical best solution of the *i*th particle and the global best solution, respectively. Besides that, *c_1_* and *c_2_* denote the position constants, while *r_1_* and *r_2_* are two random values generated from [0, 1]. Moreover, *t* and *w* represent the current iteration number and inertia weight, respectively.

### 2.2. PSO Variants

Despite its simplicity and fast convergence speed, the PSO model is subject to local optima traps and premature convergence, owing to the constant reference to the global best solution for all swarm particles. The particle positions also become increasingly similar over iterations. As such, various diversity enhancing strategies have been proposed, e.g., repulsion strategies [23], mutation operators [24], multi-swarm concepts [25,26], multiple leaders [25,27], and hybridization with other search methods [27]. Such strategies enable the search process to balance between convergence and diversity while searching for the global optimality.

Chen et al. [28] proposed a dynamic PSO model with escaping prey schemes (DPSOEP). In DPSOEP, swarm particles were categorized into three sub-swarms according to their fitness scores, i.e., ‘preys’ (top ranked particles), ‘strong particles’ (middle ranked particles), and ‘weak particles’ (lower ranked particles). The particles in the above groups subsequently followed distinctive search operations, i.e., Lévy flights, the original PSO position and a multivariate normal distribution, respectively, to search for global optimality.

Li et al. [29] proposed a multi-information fusion “triple variables with iteration” inertia weight PSO (MFTIWPSO) model, in which the inertia weight was generated using multiple information, including the particle velocity, position, random disturbance, number of iterations, as well as inertia weight score from the last iteration. The MFTIWPSO outperformed a number of baseline models for solving benchmark functions and hyper-parameter tuning in classification methods.

Wang et al. [24] proposed a diversity enhancing and neighborhood search PSO (DNSPSO) model for solving multimodal high-dimensional benchmark functions. It employed a crossover factor and a DE-based operation for trial particle generation. Moreover, a ring topology was also utilized to facilitate local and global neighborhood search operations. In addition, an eXpanded PSO (XPSO) model was proposed by Xia et al. [30], where the swarm leader and a dynamic neighboring best solution were employed to guide the social component in the PSO operation.

A distributed contribution based quantum-behaved PSO with controlled diversity (DC-QPSO) was proposed by Chen et al. [31] for solving large-scale global optimisation problems. Their model first decomposed the original problem into several sub-problems. A contribution-based mechanism was then employed to ensure more resources (i.e., more number of function evaluations) to be awarded to the sub-swarms with comparatively more fitness enhancement. A diversity control strategy based on genotype diversity (i.e., distance-based diversity) was subsequently used to increase search diversity.

Lin et al. [32] proposed an enhanced genetic learning PSO (GL-PSO) algorithm for global optimisation. In GL-PSO, the genetic operators and a ring topology were employed for the generation of fitter exemplars, which were subsequently used to guide the swarm particles.

Tan et al. [27] proposed an asynchronized learning PSO model, i.e., ALPSO, by incorporating DE, Simulated Annealing (SA) and helix search actions, for hybrid clustering and hyper-parameter fine-tuning in deep Convolutional Neural Networks (CNN) for skin lesion segmentation. Zhang et al. [33] proposed an Enhanced Sine Cosine Algorithm (SCA), which employed two randomly selected neighboring solutions and the Gaussian distribution-based search parameters for the diversification of the global best signal. Moreover, Jordehi [34] proposed an Enhanced Leader PSO (ELPSO) where a five-staged mutation mechanism (e.g., Gaussian, Cauchy and opposition-based mutations) was used for swarm leader enhancement to avoid premature convergence.

Kang et al. [35] proposed a modified PSO algorithm for optimal hyper-parameter selection of Gaussian process regression (GPR). Instead of using the inertial component as in PSO, a momentum element was proposed, which was based on the mean distance of the swarm in two successive iterations. Subsequently a mutation mechanism based on a perturbation function was proposed to further enhance the global best solution.

Yu et al. [36] developed an enhanced DE algorithm for tackling multi-objective optimisation problems. It incorporated a Gaussian mutation operator for the improvement of infeasible solutions as well as a standard DE/rand/1 operation for evolving feasible solutions according their dominance relationships.

Cao et al. [37] integrated comprehensive learning PSO (CLPSO) with an adaptive local search starting strategy to solve multimodal and CEC 2013 benchmark functions, whereas Xu et al. [38] proposed an accelerated two-stage PSO (ATPSO) method with the employment of intra-cluster distance and intra-cluster cohesion measures as objective functions, respectively, for tackling complex clustering problems. Elbaz et al. [39] developed an improved PSO-adaptive neurofuzzy inference system (ANFIS) model for the prediction of shield performance during tunneling. An improved PSO method with an adaptive inertia weight and a constriction factor was employed for the optimisation of parameters in ANFIS. The empirical results indicate that this PSO-ANFIS model offered better prediction accuracy in comparison with those of ANFIS and GA-ANFIS. Elbaz et al. [40] proposed a GA-based evolving group method of data handling (GMDH)-type neural network (GMDH-GA) model for the prediction of disc cutter life during shield tunneling. GA was adopted to identify the optimal network configurations for the GMDH-type neural network.

Besides the aforementioned studies, there are other related investigations on diversity enhancement. Among them include genetic PSO (GPSO) with a crossover operator [41], a Bare-bones PSO variant (BBPSOVA) with repulsive operations and sub-swarm mechanisms [42], a Micro-GA PSO [43], a PSO with multiple sub-swarms for multimodal function evaluation (MFOPSO) [44], and a modified PSO method (MPSOELM) with time-varying adaptive acceleration coefficients for hyper-parameter optimisation pertaining to an Extreme Learning Machine (ELM) [45].

### 2.3. PSO for Feature Selection

Feature selection methods can be broadly divided into two categories, i.e., filter and wrapper. The filter approach ranks the features individually based on certain statistical criteria, such as chi-square test [46] and mutual information [47]. The feature ranking scores indicate their relative importance to the problem. It is challenging to identify the cut-off point for selecting the most important features. Besides that, the individual-based ranking mechanisms are incapable of measuring the confounding effects of feature interactions and feature composition [1]. Instead of measuring the impact of individual features, the wrapper approach evaluates the quality of various feature subsets by taking feature interaction into account, with the learning algorithm wrapped inside. Therefore, the wrapper technique possesses interaction with classifiers to capture feature dependencies.

In addition, PSO and its variants have been widely employed as the search engines in wrapper-based feature selection methods, owing to their fast convergence speed and powerful discriminative search capabilities [3,4,10,42,43]. As an example, Gu et al. [48] proposed a Competitive Swarm Optimiser, i.e., CSO, to undertake high-dimensional feature selection tasks. In CSO, the swarm was randomly divided into two sub-swarms, and pairwise competitions were conducted between particles from each sub-swarm. The winning particle was passed on to the next generation, while the defeated particle updated its position by learning from the position of the winning particle in the cognitive component as well as the mean position of the swarm in the social component. The CSO model outperformed several existing algorithms with various initialisation strategies for discriminative feature selection.

Moradi and Gholampour [49] proposed a hybrid PSO variant, i.e., HPSO-LS, for feature selection by integrating a local search strategy into the original PSO model. Two operators, i.e., “Add” and “Delete”, were employed to enhance the local search capability of PSO. Specifically, the “Add” operator inserted the dissimilar features into the particle, while the similar features were deleted from the particle by the “Delete” operator. Evaluated with 13 classification problems, HPSO-LS significantly outperformed a number of existing dimension reduction methods. Another hybrid PSO model, i.e., HPSO-SSM, was proposed by Chen et al. [19]. Specifically, the Logistic chaotic map was used to generate the inertia weight. Subsequently, two dynamic nonlinear correction factors were employed as the search parameters in the position updating operation. A spiral search mechanism was also incorporated to increase search diversity. Evaluated with 20 UCI datasets, HPSO-SSM outperformed several feature selection methods, such as CatfishBPSO (binary PSO with catfish effect). Tan et al. [50] proposed a hybrid learning PSO model, i.e., HLPSO, to identify the most discriminative elements from the shape, color, and texture features extracted from dermoscopic images for the identification of malignant skin lesions. HLPSO adopted three probability distributions, i.e., Gaussian, Cauchy, and Levy distributions, to further enhance the top 50% promising particles. Modified FA and spiral search actions were also employed to guide the lower-ranking 50% particles. Moreover, Xue et al. [4] conducted a comprehensive review on the applications of PSO as well as other EC techniques for tackling feature selection problems.

### 2.4. Research Motivations

Table 1 depicts a detailed comparison between several existing studies (including the original PSO algorithm) and this research. The original PSO model employs a search process led by a single swarm leader. Comparatively, both proposed PSOVA1 and PSOVA2 models employ multiple hybrid global optimal signals and a number of cooperative search operations to mitigate premature convergence. In particular, PSOVA2 employs versatile search operations with diverse specified sine, cosine, and hyperbolic tangent search trajectories to overcome stagnation. Both proposed models show superior capabilities in accelerating convergence while preserving diversity, in order to mitigate premature convergence.

The research motivations of the proposed models are as follows. The classical PSO algorithm explores the search space by following single leader and the particles’ own personal best experiences, therefore lack of interactions with other neighboring elite solutions accumulate through coevolution. Owing to a monotonous search operation led by single leader, the particle positions become increasingly similar over iterations. In this research, PSOVA1 is firstly proposed to enhance local and global optimal signals through the use of neighboring historical best experiences. A set of effective cooperative search strategies is introduced to overcome the limitations of the original PSO algorithm, namely a modified PSO operation with rectified local and global best signals, spiral-based local exploitation, enhancement of the swarm leader and the worst solutions using Gaussian distributions, as well as mirroring and DE-based mutations.

Secondly, PSOVA2 is further proposed to enhance the best leader generation and the search operation embedded in PSOVA1. In particular, it employs an adaptive exemplar breeding mechanism incorporating multiple local and global best solutions to guide the search process. A new search action is also proposed by embedding diverse search coefficients yielded using sine, cosine, and hyperbolic tangent formulae. In comparison with PSOVA1 where the search mainly focuses on a modified PSO operation in principle, the aforementioned new search operations equip the search process with a variety of distinctive search behaviors and irregular search trajectories. In short, the search mechanisms in PSOVA1 and PSOVA2 work in a collaborative manner to increase search diversity and mitigate premature convergence.

Moreover, most of the aforementioned existing PSO variants employed purely the single global best solution [19,22,24,31,32,34,35,39,41,44,45,51] to guide the search process. In addition, except for a few studies such as Lin et al. [32], Srisukkham et al. [42], Tan et al. [27], and Yu et al. [36], other existing work did not adopt any exemplar breeding strategies to enhance the optimal signals or generate hybrid leaders. Although some studies adopted diverse search mechanisms [19,24,27,33,42,52], the search processes in many existing studies [22,31,32,34,35,36,39,41,44,45,51] are mainly conducted by single position updating formula. Therefore, they are more likely to suffer from premature convergence. In comparison with these existing methods, the proposed PSOVA1 and PSOVA2 models employ exemplar breeding mechanisms as well as multiple global best signals to lead the search process and avoid local optima traps. A number of position updating operations (such as local and global based search actions) are embedded in both models. When a certain search operation becomes stagnant (e.g., the global search in PSOVA1 or sine-based search in PSOVA2), the proposed models are able to adopt an alternative search action (e.g., local search in PSOVA1 or cosine-based search in PSOVA2) to drive the search out of stagnation. In addition, swarm leader and worst solution enhancement is also conducted in both methods to reduce the probabilities of being trapped in local optima. The proposed search strategies in both models work cooperatively to overcome premature convergence and increase the chances of finding global optimality.

## 3. The Proposed PSOVA1 Model

In this research, we propose two PSO variants for feature selection, which aim to overcome two major shortcomings of the original PSO model, i.e., premature convergence and weak local exploitation near the optimal solutions [4,22]. We introduce the first proposed PSO model, i.e., PSOVA1, in this section. Specifically, the proposed PSOVA1 model employs four major strategies, including (1) Gaussian distribution-based swarm leader improvement, (2) DE and mirroring schemes for worst solution enhancement, (3) a modified PSO position updating strategy based on ameliorated *pbest* and *gbest*, and (4) spiral based local exploitation. The implementation of these four mechanisms is able to increase population and search diversity, therefore increasing the likelihood of attaining global optimality as compared with the original PSO algorithm.

The novel aspects of the proposed PSOVA1 model are presented below. Firstly, we propose a modified PSO operation where the rectified forms of *gbest* and *pbest*, as well as the Logistic map-oriented chaotic inertia weight are used to increase global exploration. In particular, the personal and global best signals in the search operation are further enhanced using remote and randomly selected promising neighboring solutions to overcome stagnation. Secondly, a logarithmic spiral search mechanism oriented by *gbest* is used to intensify local exploitation. A dynamic switching probability is designed to enable the search process to balance between the aforementioned global (first) and local (second) search operations. Thirdly, Gaussian distribution is used to enhance the swarm leader. It enables *gbest* to conduct local exploitation to avoid being trapped in local optima. Then, the mirroring and DE-based mutation operations are employed to improve the three weakest particles in the swarm. The details of the proposed PSOVA1 model are illustrated in Algorithm 1.

Overall, the Gaussian distribution based *gbest* enhancement, the mutation strategies for enhancement of the worst solutions, exploration schemes assisted by ameliorated *gbest* and *pbest*, as well as the intensified fine-tuning capability using the spiral search operation, cooperate with and benefit from each other to effectively avoid being trapped in local optima and increase the likelihood of attaining global optimality. We introduce each of the four proposed strategies in detail below.
**Algorithm 1. The pseudo-code of the proposed PSOVA1 model.****1   Start** **2**   Initialise a particle swarm using the Logistic chaotic map; **3**   Evaluate each particle using the objective function *f (x)* and identify the *pbest* solution of each particle, and the global best solution, *gbest*; **4**   Construct a *Worst_memory*, which stores the three weakest particles with the lowest fitness values, and identify the worst solution as *gworst*; **5   While** (termination criteria are not met) **6   {** **7**   Conduct swarm leader enhancement using Gaussian distribution as defined in Equation (3); Use the new solution to replace *gbest* if it is fitter; **8   For** (each particle *i* in the population) **do** **9   {** **10   If** (particle *i* belongs to *Worst_memory*) **11   {** **12   If** (particle *i* is *gworst*) **13   {** **14**   Construct an offspring solution by employing the local mutation operation based on *gbest* as defined in Equation (4), and use it to replace the global worst solution if the new offspring solution is fitter; **15   Else** **16**   Construct an offspring solution by employing the DE-based mutation operation based on three randomly selected *pbest* solutions as defined in Equations (5)–(6); **17**   Evaluate the offspring solution and update the position of particle *i* in *Worst_memory* based on the annealing schedule as defined in Equation (7); **18   } End If** **19**   Update the *pbest* and *gbest* solutions; **20   } End If** **21   } End For** **22   For** (each particle *i* in the population) **do** **23   {** **24   If** Rand < *p_switch_* **25   {** **26**   Establish a memory of *group_i_* which includes all neighboring *pbest* solutions with higher or equal fitness scores than that of the *pbest* solution of the current particle *i*, i.e., *pbest_i_*; **27**   Identify the neighboring fitter *pbest* solution in *group_i_* with the highest degree of dissimilarity to *gbest*, denoted as *pbest^D^*; **28**   Calculate the ameliorated *gbest* solution, i.e., *gbest^M^*, by averaging the following two solutions, i.e., *pbest^D^* and *gbest*, as indicated in Equation (8); **29**   Randomly select another neighboring fitter *pbest* solution from *group_i_*, denoted as *pbest^R^,* **30**   Calculate the ameliorated *pbest* solution, i.e., *pbest^M^* by averaging *pbest^R^* and the personal best solution of particle *i*, *pbest_i_*, as shown in Equation (9); **31**   Conduct position updating using *gbest^M^* and *pbest^M^* for particle *i* as defined in Equation (10); **32   Else** **33**   Move particle *i* around *gbest* by following a logarithmic spiral search path as shown in Equation (11); **34   } End If** **35   } End For** **36   For** (each particle *i* in the population) **do** **37   {** **38**   Evaluate each particle *i* using the objective function; **39**   Update the *pbest* and *gbest* solutions; ****40   } End For**** **41 } End While** **42** Output *gbest*; **43**
**End**

### 3.1. A Swarm Leader Enhancing Mechanism

In the context of feature selection, both the elimination of critical features and inclusion of contradictory attributes can impose significant consequences on classification performance. Therefore, a swarm leader enhancing mechanism using the skewed Gaussian distributions is proposed to equip *gbest* with further discriminative capabilities to overcome local optima traps. Such Gaussian distributions and random walk strategies have also been widely adopted in existing studies for leader or swarm enhancement [33,34,36,50,55]. As shown in Equation (3), *gbest* is mutated successively based on three Gaussian distributions with different skewness settings. Specifically, on the basis of the *gbest* solution, the Gaussian distribution with a positive skewness (right-skewed) is likely to eliminate noisy or irrelevant features, while the operation with a negative skewness (left-skewed) is more inclined to include more discriminative features. In addition, the standard Gaussian distribution (non-skewed) is employed to conduct local exploitation of *gbest* with neutrality in determining the feature numbers [34,55,56].

(3) $$gbest_{d}^{’}=gbest_{d}+\alpha \times Gaussian\left(h\right)\times \left(U_{d}-L_{d}\right)$$

where *gbest’_d_* represents the enhanced global best solution. Parameter α denotes the step size, and is assigned as 0.1 based on the recommendation of related studies [56]. Parameter *h* represents the skewness of the Gaussian distribution, and is set as −1, 1, and 0 for the left-, right- and non-skewed Gaussian distributions, respectively, based on extensive trial-and-error processes. Besides that, *U_d_* and *L_d_* represent the upper and lower boundaries of the *d*th dimension, respectively. The new solution generated by the Gaussian distribution is used to replace *gbest* if it is fitter.

### 3.2. Mutation-Based Worst Solution Enhancement

We subsequently enhance the weak particles in the swarm by conducting the mirroring mutation on the swarm leader and a DE-based operation on the local elite solutions.

Firstly, a *gbest*-based local mutation scheme is proposed to enhance the global worst solution in the swarm. As in Equation (4), the new particle is produced by conducting the mirroring effects and reversing the sign of *gbest* with a mutation probability, *r_mu_*, in each dimension. This simulates the effects of randomly activating or de-selecting some of the features on the basis of the current best feature subset represented by *gbest*. In short, the *gbest*-based local mutation scheme guarantees a balance between preserving effective information captured by the current *gbest* solution and introducing stochastic perturbations to create new momentum for the newly generated solution. Such mirroring actions were also widely adopted in existing studies [34,57] to increase population diversity:

(4) $$x_{d}^{new}=\{\begin{array}{c} -gbest_{d}\;if\;rand\geq r_{mu},\\ gbest_{d}\;otherwise, \end{array}$$

where *r_mu_* represents the mutation probability, and is set to 0.9 based on trial-and-error and recommendations in related studies [56]. When a randomly generated value is more than or equals to *r_mu_*, the new offspring is assigned with the value of the mirroring—*gbest* solution in the *d*th dimension, otherwise it is assigned with the value of *gbest* solution in that dimension. This operation is used to yield a new offspring solution to replace the worst particle in the swarm, if it is fitter.

Secondly, a DE-based mechanism is proposed to improve the second and third worst individuals in the swarm. It produces new particles by following the mutation and crossover operations of DE using three *p_best_* solutions randomly selected from the collection of all *p_best_* individuals in the swarm, as shown in Equations (5) and (6). The differential weight, *F*, in Equation (5) is generated using the Sinusoidal chaotic map, in order to increase the variety of perturbations for the donor vector, *x^donor^_d_*, in each dimension. Furthermore, the crossover parameter, *c_r_*, is generated by the Logistic chaotic map to introduce more randomness to the crossover process in each dimension and exploit more feature interactions on a global scale. When a randomly generated value is more than *c_r_*, the current dimension in the new solution is inherited from the corresponding dimension of the personal best solution, otherwise it is inherited from that of the newly generated donor solution. Owing to the adoption of several distinctive personal best solutions in the search operations, this DE-based mutation operation is able to increase population diversity significantly when the *p_best_* solutions of the particles illustrate sufficient variance from one another in the early search stage:

(5) $$x_{d}^{donor}=pbest_{d}^{1}+F\times \left(pbest_{d}^{2}-pbest_{d}^{3}\right)$$

(6) $$x_{d}^{new}=\{\begin{array}{c} x_{d}^{donor}\;if\;rand\leq C_{r},\\ pbest_{id}\;otherwise, \end{array}$$

where *pbest^1^_d_*, *pbest^2^_d_*, and *pbest^3^_d_* represent three randomly selected *p_best_* solutions of the swarm particles in the *d*th dimension, while *pbest_i_* represents the *p_best_* solution of the current particle *i*. *x^donor^_d_* and *x^new^_d_* denote the donor and new solutions in the *d*th dimension, respectively. In addition, *F* and *c_r_* represent the differential weight and crossover factor, respectively.

The newly generated fitter solution is accepted directly while the acceptance of a weaker mutated solution is determined by an annealing schedule, as defined in Equation (7) [56]:

(7) $$p=exp\left(-\frac{\Delta f}{T}\right)>\delta$$

where *T* represents the temperature for controlling the annealing process, and Δ*f* indicates the fitness difference between the mutated and original solutions. Constant δ is a randomly generated value in the range of [0, 1]. A linear cooling schedule is employed to decrease the temperature, i.e., *T* = *σT*, whereas σ is assigned as 0.9 according to [56].

The two mutation operations based on the DE and *gbest* mirroring operations operate in parallel, in order to improve the weak particles in the swarm.

### 3.3. Diversity-Enhanced PSO Evolving Strategy

In order to address stagnations in the original PSO model, we construct two distinctive search mechanisms, i.e., a modified PSO search strategy and an intensified spiral exploitation action, to increase diversification and intensification. A dynamic switching probability schedule is also proposed to achieve the best trade-off between both mechanisms and exploit the merits of both search operations to the maximum extent.

We firstly upgrade the position updating strategy in the original PSO operation by introducing ameliorated *p_best_* and *g_best_*, combined with the Logistic chaotic map, to enhance search diversity. As indicated in Equation (8), the global best experience is ameliorated by adopting the mean position of two solutions, i.e., the *g_best_* solution and a neighboring superior *p_best_* solution, i.e., *pbest^D^*, possessing the highest degree of dissimilarity to *g_best_*. The dissimilarity measure between *g_best_* and any *p_best_* solution is determined by the number of distinctive units in their binary forms, which are converted by following the existing studies [10]. In other words, the *p_best_* solution that has the least number of shared selected features in comparison with those recommended by *g_best_* is selected as *pbest^D^*. Moreover, as defined in Equation (9), the local best experience is ameliorated by adopting the mean position of the particle’s own *p_best_* and another randomly chosen superior *p_best_* solution, i.e., *pbest^R^*, in the neighborhood. Equation (10) is used to conduct position updating, which employs the enhanced global and local optimal signals defined in Equations (8) and (9), respectively:

(8) $$gbest_{d}^{M}=\left(gbest_{d}+pbest_{d}^{D}\right)/2$$

(9) $$pbest_{d}^{M}=\left(pbest_{id}+pbest_{d}^{R}\right)/2$$

(10) $$v_{id}^{t+1}=\sigma \times v_{id}^{t}+c_{1}\times r_{1}\times \left(pbest_{d}^{M}-x_{id}^{t}\right)+c_{2}\times r_{2}\times \left(gbest_{d}^{M}-x_{id}^{t}\right)$$

where *pbest^D^* represents the *p_best_* solution with the highest degree dissimilarity to *g_best_* among all neighboring superior *p_best_* solutions, while *pbest^R^* represents a randomly chosen *p_best_* solution. Moreover, *gbest^M^* and *pbest^M^* represent the enhanced global and local optimal indicators in the proposed position updating strategy, respectively, while *σ* represents the inertia weight generated by the Logistic chaotic map.

### 3.4. An Intensified Spiral Exploitation Scheme

An intensified spiral exploitation scheme is introduced to overcome the limitations of the fine-tuning capability of the original PSO algorithm in the near optimal regions. The logarithmic spiral search is originally proposed in the MFO algorithm [11]. We employ this spiral operation to fine-tune the swarm particles in the final iterations. By conducting this local spiral search action, a search space of hyper-ellipse around *g_best_* is constructed on each dimension using the spiral function, as defined in Equations (11) and (12) [11]. As a result, the exploitation around the near-optimal solutions can be significantly intensified:

(11) $$x_{id}^{t+1}=D\times \exp\left(b\times l\right)\times \cos\left(2\pi l\right)+gbest_{d}$$

(12) $$D=\left|gbest_{d}-x_{id}^{t}\right|$$

where *D* denotes the distance between *g_best_* and particle *i* in the *d*th dimension, *b* is a constant to control the shape of logarithmic spiral, with *l* as a random number in the range of [–1, 1].

Moreover, we propose a dynamic switching probability schedule with the aim to achieve a trade-off between global exploration and local exploitation in the PSOVA1 model, as demonstrated in Equation (13):

(13) $$p_{switch}=1-{\left(iter/Max\_iter\right)}^{2}$$

where *p_switch_* denotes the switching probability, while *iter* and *Max_iter* represent the current and maximum iterations, respectively. In each iteration, when the switching probability *p_switch_* is higher than a randomly generated value in the range of [0, 1], i.e., *p_switch_ > rand*, the modified PSO operation discussed in Section 3.3 is conducted. Otherwise, the spiral search action depicted in this section is conducted. In general, the proposed dynamic schedule of *p_switch_* not only ensures sufficient global exploration to identify the promising regions in the early search stage, but also guarantees thorough exploitations in the near optimal region before converging in the final iterations.

## 4. The Proposed PSOVA2 Model

We further enhance the PSOVA1 model by incorporating new search actions accompanied with diverse nonlinear search trajectories to extend search territory. Specifically, we propose four new strategies in PSOVA2 to refine the transition between search diversity and swarm convergence, i.e., (1) an adaptive exemplar breeding mechanism incorporating multiple local and global best solutions, (2) search coefficient generation using sine, cosine, and hyperbolic tangent functions, (3) worst solution enhancement using a hybrid re-dispatching scheme, and (4) an exponential exploitation mechanism for swarm leader improvement.

PSOVA2 further strengthens PSOVA1 by providing new search mechanisms on the best leader generation and position updating operations. The novel aspects of the proposed PSOVA2 model are as follows. Firstly, an adaptive exemplar breeding mechanism is proposed, which produces a new exemplar by incorporating multiple local and global best solutions to guide the search process. On top of it, a new search action is proposed by embedding diverse search coefficients yielded using sine, cosine, and hyperbolic tangent formulae. In comparison with PSOVA1 where the search mainly focuses on a modified PSO operation in principle, the aforementioned new search operations equip the search process with a variety of distinctive search behaviors and irregular search trajectories. In addition, scattering and random permutations from the *p_best_* solutions are incorporated for enhancement of the worst solutions. An adaptive exponential search flight is also used for swarm leader improvement. These new strategies demonstrate great capabilities in accelerating convergence while preserving search diversity. The pseudo-code of PSOVA2 is provided in Algorithm 2. We introduce each proposed strategy in the following sub-sections.
**Algorithm 2. The pseudo-code of the proposed PSOVA2 model.****1   Start** **2**   Initialise a particle swarm using the Logistic chaotic map; **3**   Evaluate each particle using the objective function *f (x)* and identify the *p_best_* solution of each particle, and the global best solution, *gbest*; **4   While** (termination criteria are not met) **5   {** **6**   Conduct swarm leader enhancement as defined in Equations (26)–(27); **7**   Implement the worse solution enhancement as defined in Equations (23)–(25); **8   For** (each particle *i* in the population) **do** **9   {** **10**   Construct a breeding exemplar as defined in Equations (15)–(18); **11**   Select a coefficient generation function from Equations (19)–(22) randomly; **12   For (**each dimension *j*) **do** **13   {** % Choose the target optimal signal to follow in each dimension **14   If** Rand < 0.4 **15**   { **16**   Choose the breeding exemplar as the target signal for position updating; **17   Else** **18**   Choose the *gbest* solution as the target signal for position updating; **19   } End If** **20**   Update the position of particle *i* on dimension *j* as defined in Equation (14); **21   } End For** **22   } End For** **23   For** (each particle *i* in the population) **do** **24   {** **25**   Evaluate each particle *i* using the objective function; **26**   Update *pbest* and *gbest* solutions; **27   } End For** **28   } End While** **29**   Output *gbest*; **30   End**

### 4.1. A New Attraction Operation with Differentiated Search Trajectories

Firstly, a new search operation is proposed. It includes an exemplar breeding strategy and a search coefficient generation scheme using four nonlinear formulae. Equation (14) defines the proposed search action:

(14) $$x_{id}^{t+1}=x_{id}^{t}+f_{s}\;\times \;\left(x_{d}^{target}-x_{id}^{t}\right)\;+\;Gaussian\left(t\right)$$

where *f_s_* denotes a search coefficient generated by customized sine, cosine, and hyperbolic tangent functions, respectively, and *x^target^* represents a target optimal indicator such as the exemplar or the swarm leader. *Gaussian (t)* indicates a random walk following a Gaussian distribution. Equation (14) is used for position updating in PSOVA2. We introduce the exemplar breeding and nonlinear search coefficient generation in detail in Section 4.1.1 and Section 4.1.2, respectively.

#### 4.1.1. Exemplar Generation Using Adaptive Incorporation of Multiple Optimal Solutions

**Figure 1:**
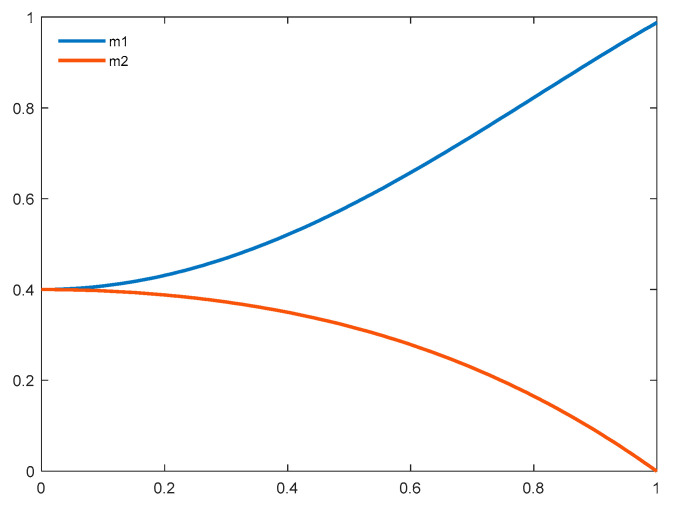
Adaptive coefficients for the *g_best_* solution (blue) and the *p_best_* signal (red) for exemplar generation (where *x* axis denotes a randomly generated value between 0 and 1, and *y* axis denotes the weight parameters, i.e., *m_1_* and *m_1_* defined in Equations (16) and (17)).

Instead of completely following the *g_best_* solution over the search course as in the original PSO algorithm, an adaptive exemplar generation scheme is proposed. It incorporates two adaptive operations for exemplar generation, i.e., (1) stochastic recombination and dynamic incorporation of different numbers of the *p_best_* elicit solutions, and (2) an adaptive weight generation to attenuate the impact imposed by the *p_best_* solutions, while amplifying the influence of the *g_best_* solution over the search course. Specifically, an exemplar is generated through the proposed breeding mechanism between the *p_best_* and *g_best_* solutions for each particle through three steps. Firstly, a predefined number of the *p_best_* solutions (i.e., three or fewer) are randomly selected, and then aggregated into one offspring solution by multiplying random but normalized weights on each dimension, as illustrated in Equation (15). Secondly, the adaptive weights for governing the priority of the aggregated offspring and the *g_best_* solution during the breeding operation are generated by two proposed mathematical formulae defined in Equations (16) and (17). Figure 1 presents a visualization of adaptive weight generation defined in Equations (16) and (17). Lastly, the exemplar solution is produced by conducting weighted aggregation between the *g_best_* solution and the offspring solution yielded by Equation (15) in each dimension, as defined in Equation (18):

(15) $$x_{d}^{offspring}=\{\begin{array}{c} (c_{1}\times pbest_{d}^{1}+c_{2}\times pbest_{d}^{2}\;+\;c_{3}\times pbest_{d}^{3})/\left(c_{1}+c_{2}+c_{3}\right)\;if\;iter\in \left[1,\;25\right],\\ (c_{1}\times pbest_{d}^{1}+c_{2}\times pbest_{d}^{2})/\left(c_{1}+c_{2}\right)\;if\;iter\in \left[26,\;50\right],\\ pbest_{d}^{1}\;if\;iter\in \left[51,\;75\right],\\ 0\;if\;iter\in \left[76,\;100\right], \end{array}$$

(16) $$m_{1}=0.4+0.5\times \sin(\pi /2\times iter/Max\_iter)\times \sinh\left(iter/Max\_iter\right)$$

(17) $$m_{2}=0.4\times \cos(\pi /2\times iter/Max\_iter)\times \cosh\left(iter/Max\_iter\right)$$

(18) $$x_{d}^{exemplar}=m_{1}\times gbest_{d}\;+\;m_{2}\times x_{d}^{offspring}$$

where *x^offspring^* and *x^exemplar^* represent the offspring solution generated from randomly sampled *p_best_* solutions and the obtained exemplar solution through the breeding mechanism, while *m_1_* and *m_2_* represent the adaptive weights for *g_best_* and *x^offspring^* respectively. Parameters *c_1_*, *c_2_*, and *c_3_* possess randomly generated scores within [0, 1].

Specifically, we prescribe a decreasing process to control the number of selected *p_best_* solutions for exemplar generation. It starts with three *p_best_* solutions being randomly selected for breeding, then eliminating one in every 25 iterations. As a result, four different cases are produced through the iterative process for *p_best_* selection, i.e., 3 for iterations [1, 25], 2 for iterations [25, 50], 1 for iterations [25, 75], and 0 for iterations [75, 100]. At the beginning of the search process, the higher number of selected *p_best_* solutions aims to introduce more perturbation to *g_best_* during breeding owing to the higher degree of optimal signal diversity and less similarity among the *p_best_* solutions. This can further facilitate the exploration in previously unexploited search territory. By eliminating the selected *p_best_* solutions through the iterative process as well as the higher similarity among elicit solutions owing to a gradually converged population, the disturbance produced by breeding on *g_best_* becomes less significant as compared with that from the early stages. Therefore, the search becomes more accelerated through the incorporation of elicit genes from *g_best_* while maintaining the necessary level of diversity owing to distinctive elements from the recombination effect among the *p_best_* solutions. When the search comes to the final stage, none of the *p_best_* solutions are selected. As such, the exemplar becomes identical to the *g_best_* solution, in order to facilitate the exploitation around the most optimal regions. As a result, the exploration at the early stage is intensified, and search diversity can be maintained through a dynamic incorporation of the *p_best_* solutions.

In addition to the above proposed mechanisms, we introduce two adaptive trajectories for regulating the impact of the *g_best_* and *p_best_* signals during breeding over the entire iterative process, as illustrated in Figure 1. The weighting factor of the *g_best_* signal (*m_1_*) keeps increasing from 0.4 to approximately 1 as the iteration increases, while that of the *p_best_* signal (*m_2_*) keeps decreasing from 0.4 to 0. Moreover, the slopes for both adaptive trajectories change slowly at the beginning of the iteration, and then gradually ascend as the number of iterations increases. As such, the impact of the *p_best_* indicators would not diminish too fast, in order to maintain diversity. At the same time, the influence of the *g_best_* solution becomes strengthened over iterations, in order to accelerate convergence. In other words, the proposed adaptive search trajectories enable the exemplar to conduct more exploration attempts in the early stage by receiving significant and diverse influence from the *p_best_* signals while ensuring a thorough exploitation around the promising regions in the final stage by receiving a dominant impact from the *g_best_* solution. As a result, the proposed trajectories are capable of accelerating convergence while preserving diversity.

The generated exemplar is subsequently used to guide the search operation. To further increase diversification and avoid stagnation, we employ diverse search coefficients yielded using sine, cosine, and hyperbolic tangent functions, which are explained in detail in the next section.

#### 4.1.2. Nonlinear Search Coefficient Generation

**Figure 2:**
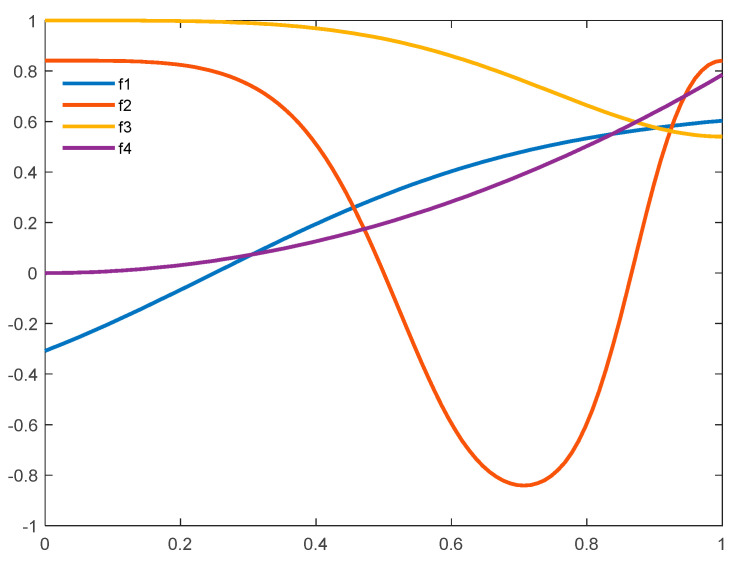
An illustration of four distinctive coefficient generation functions defined in Equations (19)–(22) (where *x* axis denotes a randomly generated value between 0 and 1, while *y* axis signifies *f_s_* as defined in Equation (14)).

We further devise four nonlinear functions for coefficient generation in Equation (14). The objective is to conduct distinctive yet complementary search operations around the exemplar and the *g_best_* solution, in order to further increase diversity and overcome stagnation. The proposed coefficient generation functions are presented in Equations (19)–(22) and plotted in Figure 2. In general, the first two functions, i.e., *f_1_* and *f_2_*, enable the particles to jump randomly in all directions around the destination optimal signal. The next two functions, i.e., *f_3_* and *f_4_*, avail the particles to approach the optimal indicator with various speeds and intensities. Specifically, as illustrated in Figure 2 (blue line), *f_1_* takes a hyperbolic tangent formula, 2/3 * tanh (2*x* – ½), as defined in Equation (19). It increases in the range of [−0.3, 0.5] in a gradual manner. It facilitates the particles to deploy a thorough exploration around the target optimal signal in two ways, i.e., approaching it slowly when positive values are generated and distancing from it mildly when negative values are yielded. In contrast, as illustrated in Figure 2 (red line), *f_1_* takes a sin (cos(2π × *rand*^2^)) formula, as defined in Equation (20). Comparing with other three functions, it changes more rapidly with a wider range approximately in [−0.9, 0.9] for coefficient generation. It enables the particles to perform larger jumps to escape from local stagnation.

As illustrated in Figure 2 (yellow line), *f_3_* takes a cos (sin(π/2 × *rand*^2^)) formula, as defined in Equation (21). It constantly maintains at a high plateau in the range of [0.5, 1]. It regulates the particles to march towards the target optimal solution with a large step, in order to accelerate convergence. On the contrary, as indicated in Figure 2 (purple line), *f_4_* takes a cos^−1^(cos(π/4 × *x*^2^)) formula, as defined in Equation (22). It increases in a gradual manner in the range of [0, 0.7]. It enables the particles to deploy an intensive exploitation in the promising regions. In each iteration, each particle is able to choose from the aforementioned four coefficient generation strategies with an equal probability to maintain search diversify. In comparison with standard sine, cosine, and hyperbolic tangent functions, the proposed refined formulae offer more erratic and irregular search trajectories:

(19) $$f_{1}=2/3*\tan h\left(2x-1/2\right)$$

(20) $$f_{2}=\sin\left(\cos\left(2\pi \times x^{2}\right)\right)$$

(21) $$f_{3}=\cos\left(\sin\left(\pi /2\times x^{2}\right)\right)$$

(22) $$f_{4}=\cos^{-1}\left(\cos\left(\pi /4\times x^{2}\right)\right)$$

where *x* is a randomly generated value within [0, 1], while *f_1_*, *f_2_*, *f_3_*, and *f_4_* are the four coefficient generation functions (i.e., specified sine, cosine, and hyperbolic tangent functions). The generated coefficients are used as search parameters *f_s_* in the position updating procedure as defined in Equation (14).

As discussed earlier, we compose these four distinctive coefficient generation strategies in a complementary manner as an effort to enhance search diversity. When the particles are trapped at local optima, large jumps and reverse directions are able to drive the search out of stagnation. On the other hand, minor movements become dominant when a detailed, near optimal exploitation is needed. Moreover, in the entire input range, the generated coefficients from these four functions are always dominated by positive signals, i.e., at least three positive outputs among four, which are able to lead the swarm to the promising regions in an accelerated manner.

The parameter generation strategies are incorporated with the proposed exemplar breeding scheme to leverage their respective advantages, i.e., diversification of the movement strategies and the destination signals. Specifically, in every position updating process, each particle is able to choose one coefficient generation function from the proposed four formulae randomly. Then, in each dimension, the particle is able to choose one best signal to follow from the breeding exemplar and the *g_best_* solution.

To be specific, the four coefficient generation strategies possess equal probabilities to be chosen for each particle. Note that *g_best_* is allocated a higher probability to be chosen when updating the particle position in each dimension, as shown in lines 8–22 in Algorithm 2. A threshold of 0.4 is determined based on trial-and-error. Such a setting is able to achieve a reasonable balance between introducing a proper perturbation and inheriting benign signals from the swarm leader.

### 4.2. A Hybrid Re-Dispatching Scheme for Enhancement of the Worst Solutions

To further accelerate convergence, we enhance several worst solutions by diverting such solutions to the optimal regions using a hybrid dispatching scheme. Specifically, we enhance the worse solutions by exploiting the personal best solutions as well as stochastic disturbance induced by random initialisation. As shown in Equations (23) and (24), two donor vectors, denoted as *x^donor1^* and *x^donor2^*, are generated by random initialisation and random permutations from the *p_best_* solutions, respectively. In particular, the element on each dimension of *x^donor2^* is obtained by inheriting the value from the corresponding dimension of a randomly selected *p_best_* solution. A random number is generated for each dimension as the determinant for the hybridization process, as shown in Equation (25). The element is inherited from the corresponding dimension of *x^donor1^* when the determinant is smaller than or equals to 0.5. Otherwise, it inherits the corresponding element from *x^donor2^*.

(23) $$x_{d}^{donor1}=L_{d}+\beta \times \left(U_{d}-L_{d}\right)$$

(24) $$x_{d}^{donor2}=pbest_{d}^{random}$$

(25) $$x_{d}^{new}=\{\begin{array}{c} x_{d}^{donor1}\;if\;rand\leq 0.5,\\ x_{d}^{donor2}\;otherwise, \end{array}$$

where *x^dono^^r1^* and *x^donor2^* represent the donor vectors generated by random initialisation and random selection from the *p_best_* solutions, respectively, while β is a random number within [0, 1]. *pbest_d_^random^* denotes a randomly selected personal best solution in the *d*-th dimension. This worst solution enhancement procedure is conducted three times to generate three offspring *x^new^_d_* solutions, which are subsequently used to replace the three worst particles with the lowest fitness scores in the swarm, respectively.

Compared with a complete random initialisation, this hybridization scheme for enhancement of the worst solutions is capable of enhancing such solutions by exploiting elicit genes from the population to accelerate convergence.

### 4.3. Swarm Leader Enhancement Using an Adaptive Exponential Search Flight

In addition, we propose an exponential function to generate random search steps for enhancement of the swarm leader, as defined in Equation (26).

**Figure 3:**
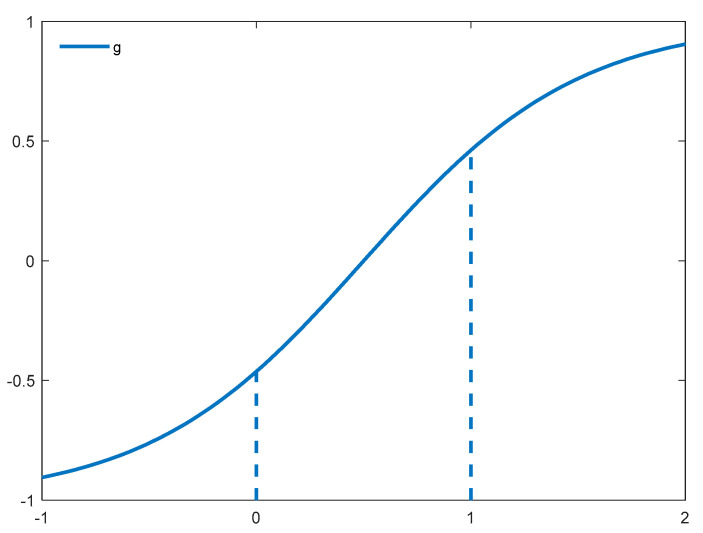
The governing function for generating the random step *g* (where *x* axis represents a randomly generated value between 0 and 1, while *y* axis signifies the search step *g*, as defined in Equation (26)):

As depicted in Figure 3, the generated step *g* is confined within [−0.5, 0.5] with an input value between [0, 1]. As a result, a smaller magnitude of steps enables the swarm leader to examine thoroughly within its vicinity from all directions, in order to discover a better position to further improve its quality. Equation (27) is used to generate an offspring solution of *gbest* using the newly generated search step *g*.

(26) $$g=2/\left(1+e^{\left(1-2x\right)}\right)-1$$

(27) $$gbest_{d}^{’}=gbest_{d}+g\times \left(U_{d}-L_{d}\right)$$

where *x* is a randomly generated value within [0, 1], while *U_d_* and *L_d_* denote the upper and lower boundaries of the *d*-th dimension, respectively. This new *gbest’* solution is used to replace *gbest* if it is fitter.

Overall, the proposed PSOVA2 model incorporates the aforementioned four major improvements to further enhance search dynamics and diversity. They include an adaptive exemplar breeding mechanism, search coefficient generation using nonlinear functions, exponential exploitation and re-dispatching schemes for swarm leader and worst solution enhancement. They account for the efficiency of PSOVA2 in accelerating convergence while maintaining diversity.

Both proposed PSO variants are integrated with a K-Nearest-Neighbor (KNN) classifier to conduct fitness evaluation during the search process. Equation (28) [3,4,42,43] defines the objective function, which is used to assess the fitness of each particle:

(28) $$fitness\left(x\right)=k_{1}\times accuracy_{x}+k_{2}\times {\left(num\_of\_features_{x}\right)}^{-1}$$

where *k_1_* and *k_2_* denote the weights of classification accuracy and the number of selected features, respectively. We assign *k_1_ = 0.9* and *k_2_* = *0.1* by following the recommendation in previous studies [3,4,42,43].

The optimisation objective of the proposed PSO models is to identify the most discriminative feature subset from a given database. The fitness function aims to maximize the classification accuracy rate while reducing the number of selected features. The search process of the most significant feature subset is conducted as follows. The particles are initialised with continuous values in each dimension using the Logistic map at the beginning of the search process. Each particle is used to represent the initial randomly assigned feature subset, where the particle dimension is the same as the number of the features in a given dataset. During fitness evaluation, we convert each element of each particle into a binary value, i.e., 1 or 0, representing the selection (1) or non-selection (0) of a particular feature. The recommended feature subset by each particle is evaluated using the training data set. The KNN model with five neighbors, as recommended in related studies [19,58], is employed to evaluate the fitness of the selected feature subset with a 10-fold cross-validation method. A fitness score is calculated using Equation (28). The identified final swarm leader represents the most optimal feature subset. We subsequently evaluate the efficiency of this selected feature subset using the unseen test set in the test phase. The aforementioned feature selection process using each proposed PSO model combining with KNN is also illustrated in Algorithm 3. We evaluate the effectiveness of both proposed PSO variants in feature selection tasks in Section 5.
**Algorithm 3. The pseudo-code of the hybrid PSOVA1/PSOVA2-KNN feature selection model.****1   Start** **2**   Initialise a particle swarm using the Logistic chaotic map; **3   For** (each particle *i* in the population) **do** **4   {** **5**   Convert particle *i* into a corresponding feature subset by selecting features on the dimensions where positive values are assigned; **6**   Calculate classification performance of the feature subset encoded in particle *i* on the training data set using the KNN classifier; **7**   Evaluate the fitness score of particle *i* based on its classification performance and number of selected features using the proposed objective function *f (x)* as shown in Equation (28); **8**   Identify the *pbest* solution of each particle and the global best solution gbest; **9   } End For** **10   While** (termination criteria are not met) **11   {** **12**   Evolve swarm particles using the proposed mechanisms in PSOVA1 (i.e., line 7–35 in **Algorithm 1**) or PSOVA2 (i.e., line 6–22 in **Algorithm 2**); **13   For** (each particle *i* in the population) **do** **14   {** **15**   Evaluate particle *i* using the objective function on the training set; **16**   Update *p_best_* and *gbest* solutions; **17   } End For** **18   } End While** **19**   Output *gbest*; **20**   Convert *gbest* into the identified optimal feature subset; **21**   Calculate classification performance on the unseen test set based on the yielded optimal feature subset using the KNN classifier; **22**   Output the test classification results & the selected features; **23   End**

## 5. Evaluation and Discussion

We employ a total of 13 datasets to investigate the efficiency of the proposed PSO models for feature selection. The employed datasets pose diverse challenges to feature selection problems, owing to a great variety of dimensionalities as well as complicated class distributions. The proposed PSO variants are integrated with a KNN-based wrapper model to conduct feature optimisation, where the number of the nearest neighbor is set to 5 according to the recommendation in previous studies [19,58]. Three performance indicators are used to examine the effectiveness of the proposed PSO variants, i.e., classification accuracy, number of selected features, and F-score. Furthermore, we compare the proposed PSO variants against five classical search algorithms, i.e., PSO [22], DE [59], SCA [60], DA [61], and GWO [62], as well as ten PSO variants, i.e., CSO [52], HPSO-SSM [19], binary PSO (BPSO) [63], modified binary PSO with local search and a swarm variability controlling scheme (MBPSO) [53], CatfishBPSO [54], GPSO [41], MPSOELM [45], MFOPSO [44], BBPSOVA [42], and ALPSO [27]. To ensure a fair comparison, we employ the same number of function evaluations (i.e., population size × the maximum number of iterations) as the stopping criterion for all search methods. In our experiments, the population size and the maximum number of iterations are set to 30 and 100, respectively, based on trial and error. We conduct 30 runs for each experiment.

### 5.1. Data Sets

We employ the ALL-IDB2 database [64] for Acute Lymphoblastic Leukaemia (ALL) diagnosis, as well as ten UCI data sets [65], namely Arcene, MicroMass, Parkinson’s disease (Parkinson), Human activity recognition (Activity), LSVT voice rehabilitation (Voice), Grammatical facial expressions (Facial Expression), Heart disease (Heart), Ionosphere, Epileptic seizure (Seizure) and Wisconsin breast cancer diagnostic data set (Wdbc), for evaluation. Besides that, two additional microarray gene expression data sets, i.e., Crohn’s disease (Crohn) and Multiple Myeloma (Myeloma), from the Gene Expression Omnibus repository [66], are employed for evaluation. The details of each data set are shown in Table 2. These data sets pose diverse challenges to feature selection models, owing to a great variety of dimensionalities and class numbers, as well as complex data distributions. Specifically, the dimensionality of the employed data sets spans from 30 to 22,283, while the number of the classes ranges from 2 to 10. Moreover, according to previous studies [42,67,68], the employed data sets contain significant challenging factors (e.g., small inter-class and large intra-class variations) which can severely affect classification performance. Overall, a comprehensive evaluation can be established for the proposed PSO variants, in view of the dimensionality, number of classes, and sample distributions, pertaining to the data sets used for evaluation.

### 5.2. Parameter Settings

We compare the proposed PSO variants against fifteen baseline methods, i.e., five classical search algorithms, i.e., PSO, DE, SCA, DA, and GWO, and ten advanced PSO variants, i.e., CSO, HPSO-SSM, BPSO, MBPSO, CatfishBPSO, GPSO, MPSOELM, MFOPSO, BBPSOVA, and ALPSO. The parameter settings of each baseline method employed in this study are set in accordance with the recommendations in their original studies. The detailed parameters of the proposed PSO models and fifteen baseline methods are presented in Table 3.

### 5.3. Results and Discussion

A comprehensive evaluation on the proposed PSO variants is established. Specifically, we adopt four different performance measures, i.e., classification accuracy, the F-score measure, number of selected features, and convergence performance, in our experiments. A total of 30 runs are conducted in each experiment, and the average results are computed for comparison. Table 4 and Table 5 summarise the classification accuracy rates, F-scores, and their corresponding standard deviation results, respectively, while Table 8 presents the numbers of selected features for all the search methods. The best results are marked in bold accordingly.

#### 5.3.1. Classification Performance

With respect to classification accuracy in Table 4, PSOVA1 and PSOVA2 achieve the highest accuracy scores on all thirteen classification tasks. They outperform all the fifteen baseline algorithms consistently. Specifically, PSOVA1 produces the highest accuracy scores on two datasets, i.e., Parkinsons and Facial Expression, while PSOVA2 yields the best accuracy scores on the remaining eleven datasets. Moreover, the empirical results reveal the advantages of the proposed models over fifteen baseline methods, especially on data sets with high dimensionalities, e.g., Crohn (22,283), Myeloma (12,625), and MicroMass (1300), as well as data sets with fuzzy boundaries and small inter-class variations, e.g., Heart (72).

Specifically, on the Heart data set, PSOVA2 outperforms the top three best classical search methods, i.e., SCA, HPSO-SSM, and DE, by 6.21%, 7.97%, and 8.06%, respectively. On the MicroMass data set, PSOVA2 outperforms the top three best search methods, i.e., GWO, BBPSOVA, and SCA, by 4.88%, 4.94%, and 5.51%, respectively. Evident performance gaps can also be observed between PSOVA1 and fifteen baseline methods. The effectiveness of both proposed PSO models is further ascertained by the F-score measure, as shown in Table 5. Similar to the accuracy measures, the proposed PSO models achieve the highest F-score performances on all thirteen data sets and outperform fifteen baseline methods with significant performance gaps, especially on feature selection tasks with high complexities, e.g., MicroMass and Heart data sets. Moreover, in comparison with those of fifteen baseline models, our proposed PSO variants demonstrate smaller or similar standard deviation results with respect to both the accuracy and F-score measures. This indicates the reliability of the proposed PSOVA1 and PSOVA2 models in producing superior classification performances across the employed feature selection tasks with various dimensionalities. The reliability of the proposed PSO variants will be further examined using the Wilcoxon statistical test.

We subsequently analyze the performance gaps pertaining to the challenges posed by some example data sets as well as the superiority of both proposed models, as follows. With respect to ALL, the proposed models have successfully identified the clinical features critical to ALL diagnosis, e.g., cytoplasm and nucleus areas, ratio between the nucleus and cytoplasm areas, form factor, compactness, perimeter, and eccentricity [42,67]. These features are commonly selected more than 15 times out of 30 trials by both proposed models. Specifically, as an important indicator of cell irregularity and eccentricity, the inclusion of the ratio between the nucleus and cytoplasm areas in the selected feature subsets can make a significant difference to accurate diagnosis of ALL. However, the baseline models often fail to consider the interactive impact between cytoplasm and nucleus owing to the negligence of either of them in the selected features. Overall, the feature selection results further indicate the effectiveness of both proposed models in identifying the most discriminatory characteristics to ALL diagnosis. In comparison, the baseline models often partially identify these important discriminative features, or overlook some aspects of sophisticated feature interactions, owing to the stagnation at local optima. Likewise, with respect to the diagnosis of coronary heart disease with three different severity levels [69], the feature subsets generated by the proposed PSO models reveal a number of key features, e.g., chest pain type, serum cholesterol, maximum heart rate, and ST depression. These have been identified as critical clinical features for the diagnosis of heart disease in existing studies [70].

The Wilcoxon rank sum test is conducted based on the mean classification accuracy rates, in order to further indicate the statistical difference of both proposed PSO models against the baseline methods. As illustrated in Table 6 and Table 7, most of the test results are lower than 0.05, ascertaining that both proposed PSO models outperform the fifteen baseline models on most of the data sets, significantly. Comparing with PSOVA1, PSOVA2 achieves a better statistical superiority. Specifically, PSOVA1 outperforms all the baseline methods for five data sets (Crohn, Myeloma, MicroMass, Parkinsons, and Activity), while PSOVA2 outperforms all the baseline methods for eight data sets (Crohn, Myeloma, Arcene, MicroMass, Parkinsons, Activity, Seizure, and Heart), with statistical significance. Out of 180 evaluations (12 data sets × 15 baseline algorithms), PSOVA1 does not show statistically significant differences in eighteen instances with respect to the baseline methods, as compared with eleven instances from PSOVA2.

The top three baseline models with the most competitive performances in comparison with those of our proposed PSO variants are DE, BBPSOVA, and SCA. Specifically, PSOVA1 shows similar result distributions to those of DE on ALL, Ionosphere, and Wdbc, to those of BBPSOVA on Voice, Ionosphere, and Wdbc, as well as to those of SCA on Arcene, Heart, and Ionosphere data sets, whereas PSOVA2 demonstrates similar performance distributions to those of DE on ALL and Wdbc, to those of BBPSOVA on Voice and Wdbc, as well as to those of SCA on Ionosphere data set.

The advantages of the proposed PSO models become more apparent on classification tasks with higher dimensionalities and sophisticated class distributions, i.e., Crohn (22,283), Myeloma (12,625), Micromass (1300), Parkinson (753), and Activity (561). PSOVA2 depicts statistically significant superiority against all the baseline methods for these high-dimensional data sets. This is because of the adoption of diverse exemplars to guide the search in each dimension, as well as the employment of versatile search trajectories to rectify particle positions.

The search strategies in most of the baseline models are monotonous, therefore are more likely to be trapped in local optima in NP-hard problems, such as feature selection tasks. Owing to the proposed comprehensive strategies of avoiding the local optima traps, the search diversity and robustness are significantly enhanced in both proposed PSO models, therefore the likelihood of ascertaining the global optima. Overall, the statistical results prove the significant superiorities of both proposed PSO models over the five classical search methods and ten advanced PSO variants, especially in feature selection tasks with higher complexities.

#### 5.3.2. Selected Feature Sizes

With respect to the number of selected features, as shown in Table 8, CSO selects the fewest numbers of features on eight data sets, i.e., Crohn, Myeloma, Arcene, Voice, Facial Expression, Seizure, ALL and Wdbc, while GWO obtains the smallest feature sizes on four data sets, i.e., MicroMass, Parkinsons, Activity, and Heart. Owing to the excessive elimination of essential features, CSO achieves the lowest classification accuracy rates on the five data sets, i.e., Micromass, Voice, ALL, Wdbc, and Crohn. This indicates that CSO falls into local optima on the above data sets during training, which leads to the stagnation in performance. According to the fitness evaluation illustrated in Equation (28), this phenomenon in turn results in the severe removal of features, in order to further improve the fitness scores. As such, very small feature subsets are identified during the feature selection process, which may not be able to capture sufficient characteristics, leading to a severe performance deterioration in the test stage. On the contrary, the proposed PSO variants succeed in achieving an efficient trade-off between eliminating redundant features and improving performance. They select comparatively smaller feature subsets than those from many search methods in most of the test cases, while achieving the highest accuracy rates and the F-score results on all thirteen test data sets. In particular, the proportions of the eliminated features by PSOVA2 are 65.46%, 66.22%, 65.88%, 63.32%, 63.51%, 66.93%, 64.84%, and 67.54%, on eight high-dimensional data sets, i.e., Crohn, Myeloma, Arcene, MicroMass, Parkinsons, Activity, Voice, and Facial Expression, respectively. A similar feature elimination capability is also depicted by PSOVA1. In short, the empirical results indicate the significant capabilities of the proposed PSO models in removing irrelevant and noisy features while identifying the most discriminative and effective ones without falling into local optima traps during the search process.

#### 5.3.3. Convergence Rates and Computational Costs

The mean convergence curves over a set of 30 runs for each search method on two high-dimensional data sets, i.e., Myeloma and Crohn, respectively, are provided to indicate model efficiency in the training stage.

**Figure 4:**
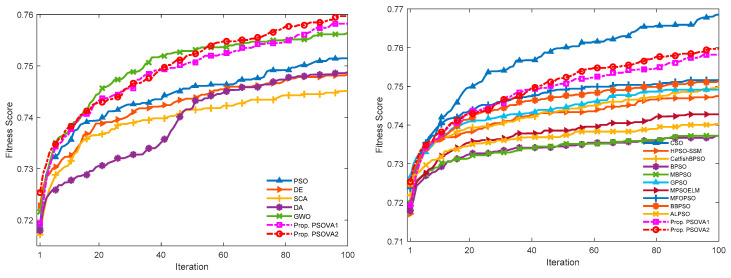
Mean convergence curves over 30 runs for the classical search methods (**left**) and advanced PSO variants (**right**) for the Multiple Myeloma data set (where *x* and *y* axes denote the iteration number and the fitness score, respectively.).

**Figure 5:**
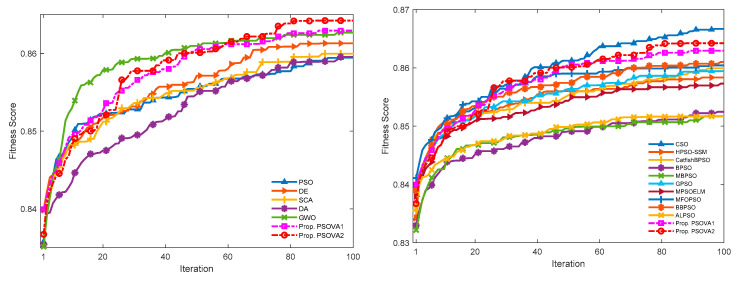
Mean convergence curves over 30 runs for the classical search methods (**left**) and advanced PSO variants (**right**) for the Crohn’s disease data set (where *x* and *y* axes denote the iteration number and the fitness score, respectively.).

As illustrated in Figure 4 (Myeloma) and Figure 5 (Crohn), both proposed PSO models (two dash lines) achieve promising results. Specifically, the proposed PSO models illustrate faster convergence rates than those from the baseline models, while maintaining the momentum to improve the fitness score through the entire search course. PSOVA2 performs better than PSOVA1, especially during the later stage of the search course. The proposed exemplar breeding mechanisms and diverse attraction operations with non-linear parameters account for the superior capabilities of PSOVA2 in preserving diversity and overcoming local stagnation. Moreover, CSO illustrates faster convergence rates than those of the proposed models, but at the expense of excessive elimination of a large number of features. It is likely that CSO is trapped in local optima, and its performance becomes stagnant. This is supported by its deterioration in classification accuracy and F-measure results, as indicated in Table 4 and Table 5. On the contrary, the proposed models achieve comparatively a balanced trade-off between feature elimination and performance improvement.

Since fitness evaluation is the most time-consuming procedure during the search cycle, the computational load of PSOVA1 and PSOVA2 primarily hinges on the population size × the maximum number of iterations. Note that all the search methods employ the same maximum number of function evaluations during the training stage. As such, all the search methods have a similar computational cost in principle, which is governed by the time taken for fitness evaluation. On the other hand, the internal search mechanisms are different from one algorithm to another, therefore the computational cost of each algorithm differs slightly. Table 9 depicts the average computational costs during training with respect to the proposed PSO models and other search methods over 30 runs on the Crohn, Myeloma, and Seizure data sets. Since they have either high-dimensional features or large sample sizes, these data sets are selected for computational cost analysis. The computational costs of all the methods pertaining to other data sets may vary in accordance with the training sample sizes and dimensions. As indicated in Table 9, in most of the cases, both proposed PSO models show comparatively lower or comparable computational costs in comparison with those from most of the baselines methods. CSO, GWO, and PSOVA2 achieve the most efficient training computational costs for Crohn, Myeloma, and Seizure data sets, respectively.

#### 5.3.4. Evaluation of The Proposed Mechanisms in PSOVA1 and PSOVA2

We subsequently demonstrate the efficiency of each proposed mechanisms in both PSOVA1 and PSOVA2 using the Seizure and Voice data sets. The mean classification accuracy rates over 30 runs are shown in Table 10. The empirical results indicate that each strategy in each proposed model is able to drive the search out of stagnation and enhance the feature selection performance. The results conform to the principles of the introduced mechanisms. In particular, the exemplar breeding mechanism and the versatile search operations using compound sine, cosine, and hyperbolic tangent functions in PSOVA2 are comparatively more effective than the modified PSO operation with ameliorated optimal signals and spiral-based local exploitation in PSOVA1. This is primarily owing to the employment of diverse exemplars to lead the search in each dimension, as well as the adoption of versatile search courses to rectify the particle positions in PSOVA2.

In comparison with the original PSO model and PSOVA1, instead of using single leader or rectified separate global and personal best experiences to guide the search process, an exemplar generation scheme with adaptive aggregation of the local and global optimal signals is used in PSOVA2. As such, the impact of the local optimal indicators is more significant at the beginning stage of the search process and the influence of the global best solution is more dominating towards the final iterations. Such an exemplar breeding scheme in PSOVA2 is more capable of overcoming stagnation. Unlike PSOVA1 where the search mainly focuses on a modified PSO algorithm, PSOVA2 employs four search strategies implemented using refined sine, cosine, and hyperbolic tangent formulae for the position updating procedure to increase search diversification.

The mechanisms proposed in both PSO models work in a collaborative manner to diversify the search process and mitigate premature convergence. In PSOVA1, when the modified PSO algorithm with rectified optimal signals becomes stagnant over the iterations, the local exploitation mechanism based on the spiral search action is able to further explore the near-optimal regions and drive the search out of stagnation. In PSOVA2, when the customized sine-based search operation is trapped in local optima, other search mechanisms such as cosine and hyperbolic tangent oriented search actions are able to extend the search territory to overcome early stagnation. In short, the empirical results indicate that the proposed mechanisms in each model offer great efficiency in mitigating premature convergence, leading to great capabilities in accelerating convergence while preserving diversity.

Besides the above, we further evaluate the efficiency of each proposed strategy in PSOVA1 and PSOVA2 for tackling minimization problems using a set of 11 benchmark functions. They include four multimodal functions (i.e., Ackley, Griewank, Rastrigin, and Powell) and seven unimodal landscapes (i.e., Dixon-Price, Rotated Hyper-Ellipsoid, Rosenbrock, Sphere, Sum of Different Powers, Sum Squares, and Zakharov). The definitions of these benchmark functions are provided in [23,34,50,71]. The following experimental settings are employed for model evaluation, i.e., population size = 30, dimension = 30, maximum number of iterations = 500, and trials = 30. Table 11 illustrates the mean, maximum, minimum, and standard deviation results for all the test functions with the best results highlighted in bold. As shown in Table 11, both the mean and minimal results over 30 runs indicate that our models with individual or composite proposed mechanisms all significantly outperform the standard PSO model. For each of the proposed PSO variants, sequential aggregation of the proposed mechanisms amounts to better search efficiencies and capabilities, as evidenced by the enhanced performances. Moreover, PSOVA2 outperforms PSOVA1 on 9 out of 11 test functions. Overall, the empirical results of the test functions demonstrate great superiority of the proposed models. The search mechanisms in PSOVA1 and PSOVA2 work in cooperation to achieve the best performances owing to the advanced trade-offs between diversification and intensification.

#### 5.3.5. Discussion

The empirical results of classification performance, feature elimination effects, as well as convergence rates all indicate the superiority of the proposed PSO variants over other baseline methods in undertaking feature selection tasks, i.e., constructing simplified but valid feature subsets while improving classification performance.

Both proposed PSOVA1 and PSOVA2 models adopt hybrid leader signals and diversified search operations to overcome local optima traps. In essence, PSOVA2 inherits all merits of PSOVA1. It further endows the particles with a higher degree of freedom in terms of (1) the choice of destination signals, and (2) the choice of movements to approach the destination solutions. Besides the generation of the combined best leader by adaptively incorporating both local and global best signals, PSOVA2 implements multiple movement operations towards the destination signal where the search coefficients are delivered by four distinctive yet complementary nonlinear functions. These search mechanisms offer the choices of either a large jump to propel the convergence or a gradual stroll to intensify the exploitation, as well as the choices of either marching towards or distancing from the destination signals. As a result, PSOVA2 is likely to attain global optimality successfully, while preventing stagnation at the local optima traps effectively.

In contrast, for the employed baseline classical search methods, certain limitations have been identified in previous studies, as widely discussed in the literature. Specifically, the search capability of DE can be severely compromised, owing to the failure of generating promising solutions within a limited number of function evaluations [56]. GWO demonstrates a strong bias towards the origin of the coordinate system attributed by its simulated model, as well as stagnation at the local optima traps owing to the poor exploration capability [72]. DA suffers from a poor exploitation capability, owing to the fact that it does not keep track of the elite solutions [2,61]. In addition, most of the existing PSO variants are equipped with improvements from the perspective of either exploration or exploitation, rather than comprehensively taking into account the trade-off between both operations. Overall, the proposed PSO models demonstrate great superiorities over the baseline methods in attaining the global optimality, owing to a delicate consideration of both global exploration and local exploitation. This is realised through distraction with the elicit solutions as well as detection with diverse steps and possible movement in all directions, respectively. Therefore, both proposed PSO models are capable of improving classification performance by identifying the most discriminative features and eliminating noisy and irrelevant ones, as evidenced by the empirical results along with the statistical tests. Moreover, PSOVA2 performs better than PSOVA1 in undertaking feature selection problems owing to the enhanced diversity induced by a greater freedom in choosing the exemplar signals to guide the search in each dimension, as well as a greater versatility in ways of approaching such destination solutions.

## 6. Conclusions

In this research, we proposed two PSO models, namely PSOVA1 and PSOVA2, for undertaking a variety of feature selection tasks. Each of the proposed models incorporates a number of distinctive search mechanisms to elevate exploitation of undiscovered search regions, guided by hybrid leader signals. These formulated strategies in each model work cooperatively to produce diverse search behaviors in terms of search flights and directions. In particular, PSOVA2 elevates search diversity by adopting adaptive exemplars as well as four search operations where the search coefficients are implemented using refined sine, cosine, and hyperbolic tangent functions to overcome stagnation.

Evaluated using a total of 13 data sets, with diverse dimensionalities from 30 to 22,283, both models outperform five classical search methods and ten advanced PSO variants significantly in most test cases, as evidenced by the empirical and statistical test results. Specifically, PSOVA1 outperforms all the baseline methods for five data sets (Crohn, Myeloma, MicroMass, Parkinsons, and Activity), while PSOVA2 outperforms all the baseline methods for eight data sets (Crohn, Myeloma, Arcene, MicroMass, Parkinsons, Activity, Seizure and Heart), with statistical significance.

In future directions, other hybrid leader breeding mechanisms will be explored to further enhance performance. Moreover, we also aim to evaluate the proposed models using complex computer vision tasks, e.g., deep architecture generation for object detection and classification [51,73,74,75] as well as image description generation [76,77].

## Figures and Tables

**Table 1:** Comparison between existing studies and this research.

Studies	Population Initialisation	Multiple Leaders	Exemplar Breeding Strategies	Modification of Existing Search Operations	Novel Search Mechanisms	Leader Enhancement	Other Diversity Enhancing Strategies
PSO [22]	Random	No (single leader)	No	No (the original PSO operation)	No	No	No
Wang et al. [24]	Random	No	No	No	Local and global neighborhood search based on the ring topology	No	Trial particle generation using a crossover factor & a DE operation
Lin et al. [32]	Random	No	Ring topology for exemplar generation	The updated PSO operation with the exemplar and the adaptive parameters	No	No	No
Chen et al. [31]	Random	No	No	Expansion-contraction coefficient and diversity measurement used in position updating	No	No	Genotype diversity measure and contribution-based fitness evaluation allocation
Chang [44] (MFOPSO)	Random	No	No	The search led by each sub-swarm leader	No	No	Multiple sub-swarms
Fielding et al. [51]	Random	No	No	Cosine-based adaptive search parameters	No	No	No
Srisukkham et al. [42] (BBPSOVA)	Random	The mean of all the personal bests	The average of the local and global best solutions	The average of the local and global optimal signals leading the attraction action	An evading action led by the mean of the worst indicators	No	Two sub-swarms
Tan et al. [27] (ALPSO)	Random	Two remote swarm leaders	The best leader and a remote second leader	Using helix search coefficients	Hybridization with SA and DE operations	No	No
Chen et al. [41] (GPSO)	Random	No	No	No	No	No	A crossover operator for population diversification
Nayak et al. [45] (MPSOELM)	Random	No	No	Using time-varying acceleration coefficients and an adaptive inertia weight	No	No	No
Jordehi [34] (ELPSO)	Random	No	No	No	No	5-staged mutation	No
Kang et al. [35]	Random	No	No	A momentum element is used to replace the inertial component.	No	Mutation-based leader enhancement	No
Zhang et al. [33]	Random	No	No	No	Local search action using two randomly selected particles with a Gaussian search step	No	Distance-based population diversity estimation
Yu et al. [36]	Random	No	Solution selection based on domination relationships and density measurement	No	No	No	Infeasible solution enhancement using Gaussian mutation
Chen et al. [19] (HPSO-SSM)	Random	No	No	Using a logistic map to generate the inertia weight	Local exploitation using a spiral search operation	No	Nonlinear coefficients used for velocity updating
Cheng and Jin [52] (CSO)	Random	Winners from pairwise competition	No	Using a logarithmic linear regression relationship to generate the coefficient for the social component	Position updating by learning from the winner solution	No	No
Vieira et al. [53] (MBPSO)	Random	No	No	No	Resetting the swarm leader by deselecting features, and mutation on personal best solutions by flipping randomly		Using a mirroring operation when the maximum velocity is reached
Chuang et al. [54] (CatfishBPSO)	Random	No	No	No	10% worst solutions replaced by dimension-wise random assignment	No	No
Elbaz et al. [39]	Random	No	No	Using a time-varying adaptive inertia weight and a constriction factor for velocity updating	No	No	No
PSOVA1 (This research)	Logistic map	An enhanced hybrid global best signal	Enhancing local and global best solutions using neighboring personal best experiences	The updated PSO operation with enhanced local and global best signals.	Local exploitation using a spiral search operation	Swarm leader enhancement using Gaussian distributions	Mutation and DE-based worst solution enhancement
PSOVA2 (This research)	Logistic map	An adaptive exemplar incorporating multiple local and global best solutions	Exemplar generation using adaptive weightings between local and global optimal signals, as well as a dynamic number of local best solutions.	N/A	A new search operation using the exemplar or the swarm leader as the best signal, with search coefficients generated using sine, cosine and hyperbolic tangent functions.	Swarm leader enhancement using an adaptive exponential function	Worst solution enhancement using a hybrid re-dispatching scheme

**Table 2:** Introduction of the thirteen data sets for evaluation.

Data Set	Number of Attributes	Number of Classes	Number of Instances
Crohn	22,283	2	127
Myeloma	12,625	2	173
Arcene	10,000	2	200
MicroMass	1300	10	360
Parkinsons	753	2	756
Activity	561	6	1000
Voice	310	2	126
Facial Expression	301	2	1062
Seizure	178	2	4600
ALL	80	2	180
Heart	72	4	124
Ionosphere	33	2	253
Wdbc	30	2	569

**Table 3:** Parameter settings of each algorithm.

Algorithm	Parameters
PSO [22]	cognitive component $c_{1}$ = 2, social component $c_{2}$ = 2, inertial weight w = 0.9−m×((0.9−0.4)/max_iter, where m and max_iter denote the current and maximum iteration numbers, respectively.
DE [59]	differential weight F ∈(0, 1), crossover parameter $C_{r}$ = 0.4.
SCA [60]	$r_{1}=a-m\times a/max\_iter$, where a=3. $r_{2}=2\pi \times rand$, $r_{3}=2\times rand$, and $r_{4}=rand.$$r_{1}$, $r_{2}$, $r_{3}$ and $r_{4}$ are four main search parameters.
DA [61]	separation factor = 0.1, alignment factor = 0.1, cohesion factor = 0.7, food factor = 1, enemy factor = 1, inertial weight = 0.9−m×((0.9−0.4)/max_iter.
GWO [62]	$A=2\times a\times r_{1}-a$, where a is linearly decreasing from 2 to 0, and $r_{1}\in \left(0,\;1\right)$. $C=2\times r_{2}$, where $r_{2}\in \left(0,\;1\right)$. A and C are both coefficient vectors.
CSO [52]	$r_{1}$, $r_{2}$, $r_{3}$∈(0, 1), where $r_{1}$, $r_{2}$, and $r_{3}$ are search parameters randomly selected within [0, 1]. controlling parameter Φ = 0.1.
HPSO-SSM [19]	cognitive component $c_{1}$ = 2, social component $c_{2}$ = 2, inertial weight w = Logistic map. $R_{1}=1/(1+exp{\left(a\times \left(-\min\left(SP\right)/\max\left(SP\right)\right)\right)}^{t}$, where SP is the particle position vector, while t is the current iteration, and a=2. $R_{2}=1-R_{1}$.
BPSO [63]	cognitive component $c_{1}$ = 2, social component $c_{2}$ = 2, $w_{max}$ = 0.9, $w_{min}$ = 0.01, inertial weight w = $w_{max}-m\times \left(w_{max}-w_{min}\right)/max\_iter$.
MBPSO [53]	cognitive component $c_{1}$ = 2, social component $c_{2}$ = 2, inertial weight w = 1.4, mutation probability $r_{mu}=1/N_{t}$, where $N_{t}$ represents the dimensionality of the problem domain.
CatfishBPSO [54]	cognitive component $c_{1}$ = 2, social component $c_{2}$ = 2, inertial weight w = 1, replacing rate = 0.1.
GPSO [41]	inertia weight = 0.9, cognitive component $c_{1}\;$= 2.6, social component $c_{2}\;$= 1.5, crossover probability = 0.7, mutation probability = 0.3.
MPSOELM [45]	time-varying acceleration coefficients and an adaptive inertia weight.
MFOPSO [44]	inertia weight=0.9, cognitive component $c_{1}\;$= 2, social component $c_{2}\;$= 2.
BBPSOVA [42]	search coefficients yielded by Logistic map.
ALPSO [27]	inertia weight=0.6, search parameters produced by helix functions.
Prop. PSOVA1	cognitive component $c_{1}$ = 2, social component $c_{2}$ = 2, inertial weight w = Logistic map, mutation probability threshold $r_{mu}$ = 0.9, F = Sinusoidal map.
Prop. PSOVA2	switching probability for exemplar adoption = 0.4, initial weight for $g_{best}$ = 0.4, search coefficients implemented using exponential, sine, cosine, and hyperbolic tangent functions.

**Table 4:** The mean results of the classification accuracy rates over 30 runs.

Data Sets	Metrics	PSO	DE	SCA	DA	GWO	CSO	HPSO-SSM	Catfish-BPSO	Prop. PSOVA1	Prop. PSOVA2
Crohn	mean	0.7556	0.7624	0.7479	0.7427	0.7786	0.7197	0.7675	0.7803	0.8128	**0.8333**
	std.	6.74E-02	3.10E-02	3.18E-02	3.28E-02	3.07E-02	7.16E-02	3.10E-02	3.73E-02	2.90E-02	3.09E-02
Myeloma	mean	0.7096	0.7288	0.7013	0.7032	0.7212	0.6917	0.7128	0.6910	0.7442	**0.7545**
	std.	2.60E-02	2.29E-02	2.03E-02	2.42E-02	2.37E-02	6.01E-02	2.48E-02	1.56E-02	2.68E-02	2.66E-02
Arcene	mean	0.7217	0.7244	0.7372	0.7183	0.7211	0.7372	0.7122	0.7100	0.7411	**0.7694**
	std.	2.66E-02	2.78E-02	3.98E-02	3.71E-02	2.95E-02	3.79E-02	3.28E-02	3.77E-02	2.81E-02	3.58E-02
MicroMass	mean	0.5897	0.6052	0.6061	0.5933	0.6124	0.5409	0.5903	0.5836	0.6455	**0.6612**
	std.	4.34E-02	3.85E-02	5.13E-02	4.07E-02	4.38E-02	2.79E-02	4.12E-02	3.92E-02	4.59E-02	4.38E-02
Parkinsons	mean	0.7949	0.7990	0.7922	0.7862	0.7940	0.7985	0.8000	0.7994	**0.8115**	0.8094
	std.	1.74E-02	1.63E-02	2.48E-02	2.15E-02	1.91E-02	1.30E-02	1.77E-02	1.56E-02	1.88E-02	1.60E-02
Activity	mean	0.8813	0.8919	0.8826	0.8785	0.8929	0.8876	0.8860	0.8785	0.9025	**0.9117**
	std.	1.64E-02	1.55E-02	1.86E-02	2.23E-02	1.44E-02	1.60E-02	1.95E-02	1.42E-02	1.28E-02	1.53E-02
Voice	mean	0.8237	0.8149	0.8202	0.8272	0.8219	0.7789	0.8237	0.8193	0.8526	**0.8632**
	std.	5.00E-02	5.58E-02	4.66E-02	5.83E-02	5.42E-02	8.37E-02	5.09E-02	3.95E-02	4.28E-02	4.37E-02
Facial Expression	mean	0.7187	0.6748	0.6891	0.6635	0.6844	0.6861	0.6914	0.6998	**0.7351**	0.7340
	std.	4.64E-02	4.70E-02	4.05E-02	3.37E-02	4.68E-02	5.14E-02	3.86E-02	4.21E-02	4.60E-02	4.24E-02
Seizure	mean	0.8459	0.8590	0.8543	0.8577	0.8655	0.8490	0.8461	0.8516	0.8698	**0.8860**
	std.	5.08E-03	6.69E-03	1.12E-02	1.00E-02	2.01E-02	9.22E-03	5.28E-03	8.12E-03	5.13E-03	6.12E-03
ALL	mean	0.8951	0.9167	0.9037	0.9025	0.8858	0.8728	0.8944	0.9123	0.9185	**0.9241**
	std.	2.84E-02	2.69E-02	2.21E-02	1.91E-02	4.25E-02	5.59E-02	4.76E-02	3.28E-02	3.23E-02	3.26E-02
Heart	mean	0.5963	0.6435	0.6620	0.5537	0.6398	0.5713	0.6444	0.5769	0.6731	**0.7241**
	std.	8.33E-02	5.18E-02	5.56E-02	6.13E-02	6.35E-02	4.34E-02	4.83E-02	7.16E-02	4.63E-02	5.42E-02
Ionosphere	mean	0.8171	0.8285	0.8320	0.8101	0.8197	0.8184	0.8189	0.8066	0.8351	**0.8434**
	std.	2.70E-02	3.10E-02	2.94E-02	2.62E-02	2.28E-02	2.89E-02	2.60E-02	2.89E-02	2.49E-02	2.16E-02
Wdbc	mean	0.9520	0.9534	0.9191	0.9458	0.9386	0.8828	0.9261	0.9497	0.9571	**0.9585**
	std.	1.04E-02	1.60E-02	4.19E-02	2.36E-02	3.30E-02	3.33E-02	3.60E-02	1.67E-02	1.33E-02	9.59E-03
Data sets	Metrics	BPSO		MBPSO	GPSO	MPSO-ELM	MFO-PSO	BBPSO-VA	ALPSO	Prop. PSOVA1	Prop. PSOVA2
Crohn	mean	0.7427		0.7795	0.7504	0.7479	0.7726	0.7684	0.7889	0.8128	**0.8333**
	std.	3.00E-02		2.25E-02	1.86E-02	3.45E-02	3.67E-02	3.00E-02	3.08E-02	2.90E-02	3.09E-02
Myeloma	mean	0.6942		0.7051	0.7045	0.6917	0.7154	0.7128	0.7051	0.7442	**0.7545**
	std.	2.13E-02		1.94E-02	2.30E-02	2.56E-02	2.24E-02	2.81E-02	1.94E-02	2.68E-02	2.66E-02
Arcene	mean	0.7111		0.7117	0.7022	0.7106	0.7372	0.7200	0.7161	0.7411	**0.7694**
	std.	3.53E-02		2.79E-02	3.54E-02	3.18E-02	3.62E-02	4.23E-02	2.80E-02	2.81E-02	3.58E-02
MicroMass	mean	0.5758		0.5785	0.6052	0.5879	0.5915	0.6118	0.5994	0.6455	**0.6612**
	std.	3.58E-02		4.01E-02	3.46E-02	4.31E-02	4.77E-02	3.99E-02	5.30E-02	4.59E-02	4.38E-02
Parkinsons	mean	0.7988		0.7962	0.7953	0.7890	0.7822	0.7907	0.7950	**0.8115**	0.8094
	std.	1.97E-02		1.97E-02	1.85E-02	1.84E-02	2.49E-02	1.97E-02	2.00E-02	1.88E-02	1.60E-02
Activity	mean	0.8725		0.8775	0.8864	0.8785	0.8806	0.8848	0.8810	0.9025	**0.9117**
	std.	1.59E-02		1.15E-02	1.23E-02	1.54E-02	1.80E-02	1.52E-02	1.74E-02	1.28E-02	1.53E-02
Voice	mean	0.8263		0.8246	0.8526	0.8298	0.8377	0.8439	0.8175	0.8526	**0.8632**
	std.	4.43E-02		4.72E-02	5.07E-02	3.83E-02	7.03E-02	5.72E-02	6.19E-02	4.28E-02	4.37E-02
Facial Expression	mean	0.7170		0.7274	0.7177	0.7234	0.7031	0.7061	0.7032	**0.7351**	0.7340
	std.	3.56E-02		3.93E-02	4.33E-02	4.67E-02	5.89E-02	4.62E-02	4.40E-02	4.60E-02	4.24E-02
Seizure	mean	0.8370		0.8388	0.8492	0.8400	0.8519	0.8496	0.8430	0.8698	**0.8860**
	std.	4.74E-03		4.41E-03	5.62E-03	5.84E-03	5.14E-03	6.87E-03	7.06E-03	5.13E-03	6.12E-03
ALL	mean	0.8938		0.8988	0.9068	0.8981	0.9000	0.9019	0.9025	0.9185	**0.9241**
	std.	1.97E-02		3.32E-02	2.68E-02	2.78E-02	2.64E-02	2.25E-02	2.62E-02	3.23E-02	3.26E-02
Heart	mean	0.5815		0.5750	0.5944	0.5991	0.6426	0.6250	0.6333	0.6731	**0.7241**
	std.	5.91E-02		6.50E-02	5.67E-02	7.30E-02	8.86E-02	6.87E-02	7.29E-02	4.63E-02	5.42E-02
Ionosphere	mean	0.8276		0.8110	0.8189	0.8140	0.8171	0.8228	0.8197	0.8351	**0.8434**
	std.	2.60E-02		3.27E-02	2.03E-02	3.18E-02	2.83E-02	2.30E-02	2.26E-02	2.49E-02	2.16E-02
Wdbc	mean	0.9501		0.9454	0.9517	0.9481	0.9509	0.9540	0.9501	0.9571	**0.9585**
	std.	1.10E-02		2.12E-02	9.18E-03	1.63E-02	1.31E-02	1.06E-02	1.24E-02	1.33E-02	9.59E-03

**Table 5:** The mean results of the F-score measures over 30 runs.

Data Sets	Metrics	PSO	DE	SCA	DA	GWO	CSO	HPSO-SSM	Catfish-BPSO	Prop. PSOVA1	Prop. PSOVA2
Crohn	mean	0.8202	0.8052	0.7943	0.7906	0.8236	0.7765	0.8137	0.8263	0.8550	**0.8585**
	std.	3.69E-02	2.42E-02	2.44E-02	2.52E-02	2.42E-02	5.71E-02	2.39E-02	2.90E-02	2.24E-02	2.43E-02
Myeloma	mean	0.8219	0.8411	0.8091	0.8105	0.8286	0.8026	0.8229	0.8034	0.8500	**0.8551**
	std.	1.64E-02	1.45E-02	1.27E-02	1.47E-02	1.40E-02	4.68E-02	1.58E-02	1.01E-02	1.57E-02	1.63E-02
Arcene	mean	0.6759	0.6757	0.6963	0.6780	0.6783	0.6959	0.6646	0.6574	0.6977	**0.7130**
	std.	3.85E-02	3.87E-02	4.94E-02	4.89E-02	3.31E-02	5.40E-02	4.60E-02	5.14E-02	3.16E-02	4.18E-02
MicroMass	mean	0.6349	0.6469	0.6428	0.6314	0.6445	0.5982	0.6350	0.6275	0.6759	**0.6967**
	std.	4.26E-02	3.48E-02	4.79E-02	3.94E-02	4.36E-02	2.17E-02	4.21E-02	3.88E-02	4.19E-02	4.03E-02
Parkinsons	mean	0.8691	0.8712	0.8670	0.8631	0.8686	0.8701	0.8720	0.8719	**0.8798**	0.8783
	std.	1.15E-02	1.10E-02	1.73E-02	1.41E-02	1.33E-02	8.93E-03	1.13E-02	1.01E-02	1.32E-02	1.02E-02
Activity	mean	0.8864	0.8962	0.8874	0.8833	0.8971	0.8930	0.8901	0.8838	0.9067	**0.9131**
	std.	1.53E-02	1.49E-02	1.76E-02	2.16E-02	1.37E-02	1.65E-02	1.90E-02	1.34E-02	1.24E-02	1.44E-02
Voice	mean	0.7180	0.7381	0.7265	0.7316	0.7208	0.6890	0.7339	0.7328	0.7764	**0.7852**
	std.	9.23E-02	7.09E-02	8.03E-02	1.07E-01	9.79E-02	1.06E-01	8.13E-02	7.23E-02	6.94E-02	7.54E-02
Facial Expression	mean	0.6458	0.6191	0.6288	0.6175	0.6287	0.5670	0.6292	0.6342	**0.6572**	0.6562
	std.	3.18E-02	3.10E-02	2.51E-02	1.86E-02	3.14E-02	1.92E-01	2.54E-02	2.81E-02	3.02E-02	2.87E-02
Seizure	mean	0.8197	0.8384	0.8359	0.8364	0.8486	0.8434	0.8199	0.8279	0.8526	**0.8759**
	std.	7.33E-03	8.96E-03	1.50E-02	1.41E-02	2.90E-02	9.36E-03	8.21E-03	1.11E-02	8.08E-03	8.76E-03
ALL	mean	0.9204	0.9345	0.9250	0.9266	0.9084	0.9037	0.9168	0.9331	0.9361	**0.9408**
	std.	2.28E-02	2.17E-02	1.62E-02	1.37E-02	3.93E-02	4.34E-02	4.37E-02	2.51E-02	2.67E-02	2.60E-02
Heart	mean	0.6039	0.6502	0.6661	0.5616	0.6436	0.5823	0.6513	0.5881	0.6783	**0.7271**
	std.	8.59E-02	5.25E-02	5.68E-02	6.81E-02	6.72E-02	4.53E-02	4.88E-02	7.28E-02	4.63E-02	5.49E-02
Ionosphere	mean	0.8439	0.8516	0.8550	0.8375	0.8427	0.8418	0.8452	0.8371	0.8562	**0.8625**
	std.	2.06E-02	2.48E-02	2.33E-02	2.04E-02	2.23E-02	2.52E-02	2.05E-02	2.18E-02	2.05E-02	1.77E-02
Wdbc	mean	0.9340	0.9355	0.8836	0.9246	0.9146	0.8286	0.8957	0.9308	0.9415	**0.9432**
	std.	1.47E-02	2.34E-02	6.53E-02	3.57E-02	4.84E-02	5.04E-02	5.38E-02	2.47E-02	1.94E-02	1.31E-02
Data sets	Metrics	BPSO		MBPSO	GPSO	MPSO-ELM	MFO-PSO	BBPSO-VA	ALPSO	Prop. PSOVA1	Prop. PSOVA2
Crohn	mean	0.7889		0.8220	0.7945	0.7937	0.8188	0.8153	0.8306	0.8550	**0.8585**
	std.	2.19E-02		1.68E-02	1.35E-02	2.53E-02	2.89E-02	2.29E-02	2.40E-02	2.24E-02	2.43E-02
Myeloma	mean	0.8057		0.8189	0.8186	0.8031	0.8248	0.8234	0.8189	0.8500	**0.8551**
	std.	1.32E-02		1.23E-02	1.41E-02	1.58E-02	1.39E-02	1.74E-02	1.22E-02	1.57E-02	1.63E-02
Arcene	mean	0.6573		0.6590	0.6460	0.6602	0.6985	0.6732	0.6673	0.6977	**0.7130**
	std.	4.56E-02		3.58E-02	4.65E-02	4.13E-02	4.78E-02	5.20E-02	2.36E-02	3.16E-02	4.18E-02
MicroMass	mean	0.6219		0.6200	0.6451	0.6360	0.6308	0.6449	0.6444	0.6759	**0.6967**
	std.	4.05E-02		3.59E-02	3.05E-02	3.95E-02	4.30E-02	3.92E-02	5.00E-02	4.19E-02	4.03E-02
Parkinsons	mean	0.8716		0.8702	0.8695	0.8656	0.8612	0.8662	0.8688	**0.8798**	0.8783
	std.	1.30E-02		1.31E-02	1.26E-02	1.22E-02	1.70E-02	1.32E-02	5.02E-02	1.32E-02	1.02E-02
Activity	mean	0.8783		0.8824	0.8913	0.8842	0.8854	0.8895	0.8854	0.9067	**0.9131**
	std.	1.55E-02		1.14E-02	1.16E-02	1.47E-02	1.61E-02	1.45E-02	1.70E-02	1.24E-02	1.44E-02
Voice	mean	0.7368		0.7399	0.7804	0.7398	0.7598	0.7656	0.7272	0.7764	**0.7852**
	std.	7.58E-02		7.76E-02	7.78E-02	6.51E-02	1.06E-01	8.83E-02	5.07E-02	6.94E-02	7.54E-02
Facial Expression	mean	0.6527		0.6556	0.6372	0.6537	0.6404	0.6360	0.6371	**0.6572**	0.6562
	std.	2.63E-02		2.93E-02	3.04E-02	3.50E-02	4.15E-02	2.97E-02	4.81E-02	3.02E-02	2.87E-02
Seizure	mean	0.8066		0.8094	0.8243	0.8111	0.8282	0.8251	0.8155	0.8526	**0.8759**
	std.	6.61E-03		6.33E-03	7.72E-03	8.31E-03	6.98E-03	9.39E-03	9.87E-03	8.08E-03	8.76E-03
ALL	mean	0.9195		0.9241	0.9283	0.9237	0.9244	0.9215	0.9253	0.9361	**0.9408**
	std.	1.55E-02		2.44E-02	2.07E-02	2.02E-02	1.96E-02	1.94E-02	3.53E-02	2.67E-02	2.60E-02
Heart	mean	0.5904		0.5788	0.6006	0.6166	0.6442	0.6319	0.6381	0.6783	**0.7271**
	std.	6.62E-02		7.86E-02	6.33E-02	7.14E-02	8.75E-02	6.94E-02	7.52E-02	4.63E-02	5.49E-02
Ionosphere	mean	0.8521		0.8380	0.8452	0.8419	0.8426	0.8476	0.8453	0.8562	**0.8625**
	std.	1.82E-02		2.51E-02	1.50E-02	2.44E-02	2.45E-02	1.89E-02	3.31E-02	2.05E-02	1.77E-02
Wdbc	mean	0.9312		0.9239	0.9338	0.9286	0.9325	0.9366	0.9321	0.9415	**0.9432**
	std.	1.55E-02		3.10E-02	1.29E-02	2.33E-02	1.85E-02	1.53E-02	9.79E-03	1.94E-02	1.31E-02

**Table 6:** The Wilcoxon rank sum test results of the proposed PSOVA1 model.

Data Sets	PSO	DE	SCA	DA	GWO	CSO	HPSO-SSM	Catfish-BPSO
Crohn	2.25E-04	4.45E-07	5.04E-09	4.90E-10	9.82E-05	4.42E-08	2.14E-06	6.80E-04
Myeloma	1.35E-05	3.40E-02	1.25E-07	1.26E-06	9.24E-04	8.59E-05	7.27E-05	6.63E-10
Arcene	1.53E-02	3.53E-02	**8.75E-01**	2.44E-02	1.93E-02	**6.16E-01**	1.48E-03	4.41E-04
MicroMass	2.47E-04	7.55E-03	8.69E-03	3.50E-04	4.11E-02	1.05E-09	2.12E-04	2.90E-05
Parkinsons	1.65E-03	3.15E-02	6.60E-03	1.99E-05	2.38E-03	3.35E-02	4.69E-02	4.52E-02
Activity	3.93E-06	6.61E-03	1.27E-04	1.40E-05	1.19E-02	4.51E-05	1.05E-03	1.49E-07
Voice	3.21E-02	6.20E-03	9.98E-03	4.48E-02	2.78E-02	9.85E-04	3.35E-02	4.04E-03
Facial Expression	**5.24E-01**	8.72E-05	1.23E-03	1.75E-06	5.63E-04	4.14E-05	5.06E-04	4.69E-03
Seizure	3.07E-11	1.16E-03	3.33E-05	2.05E-04	**5.49E-01**	1.23E-08	7.52E-11	1.23E-07
ALL	7.85E-03	**7.75E-01**	4.79E-02	2.92E-02	3.45E-03	1.35E-03	3.82E-02	**4.76E-01**
Heart	1.44E-04	2.16E-02	**2.94E-01**	2.20E-09	3.15E-02	1.21E-09	3.84E-02	1.29E-06
Ionosphere	1.16E-02	**6.10E-01**	**8.11E-01**	1.15E-03	4.18E-02	3.82E-02	2.77E-02	2.06E-04
Wdbc	2.48E-02	**5.23E-01**	3.02E-05	1.30E-02	3.54E-02	5.44E-09	1.84E-04	1.84E-02
Data sets	BPSO		MBPSO	GPSO	MPSOELM	MFOPSO	BBPSOVA	ALPSO
Crohn	1.45E-09		2.47E-05	2.25E-10	7.14E-09	2.94E-05	3.11E-06	4.13E-03
Myeloma	1.44E-08		5.89E-07	1.00E-06	2.53E-08	1.14E-04	1.73E-04	4.49E-07
Arcene	6.08E-04		6.28E-04	5.12E-05	6.38E-04	**8.34E-01**	2.15E-02	3.18E-03
MicroMass	5.30E-06		1.13E-05	5.31E-03	1.38E-04	6.88E-04	1.99E-02	4.66E-03
Parkinsons	3.93E-02		3.31E-02	4.74E-03	4.31E-04	4.41E-05	3.81E-04	6.72E-03
Activity	1.07E-08		2.12E-08	3.99E-05	2.71E-07	6.55E-06	4.71E-05	9.65E-06
Voice	2.91E-02		1.83E-02	**8.87E-01**	3.84E-02	**4.52E-01**	**6.03E-01**	1.61E-02
Facial Expression	1.92E-02		**3.40E-01**	3.56E-02	**3.28E-01**	4.65E-02	1.45E-02	4.03E-02
Seizure	2.92E-11		2.91E-11	4.85E-10	2.89E-11	3.40E-09	2.96E-09	4.62E-11
ALL	1.98E-03		3.11E-02	1.29E-01	1.77E-02	2.38E-02	3.03E-02	4.75E-02
Heart	2.87E-07		1.26E-07	1.56E-06	4.88E-05	4.85E-02	2.28E-03	9.35E-03
Ionosphere	**7.87E-01**		4.58E-03	2.04E-02	3.37E-02	2.40E-02	**1.22E-01**	3.59E-02
Wdbc	1.82E-02		4.16E-03	2.61E-02	4.01E-02	4.90E-02	**2.13E-01**	4.50E-02

**Table 7:** The Wilcoxon rank sum test results of the proposed PSOVA2 model.

Data Sets	PSO	DE	SCA	DA	GWO	CSO	HPSO-SSM	Catfish-BPSO
Crohn	5.69E-05	2.58E-07	4.33E-09	8.51E-10	4.23E-05	2.57E-08	1.19E-06	2.44E-04
Myeloma	2.30E-07	5.30E-04	1.13E-09	1.12E-08	8.27E-06	2.13E-06	6.73E-07	5.56E-11
Arcene	1.83E-06	5.23E-06	1.85E-03	3.00E-06	2.25E-06	7.92E-04	6.24E-07	1.48E-06
MicroMass	1.43E-06	4.26E-05	2.06E-04	6.32E-06	6.27E-04	5.29E-11	2.36E-06	3.98E-07
Parkinsons	1.48E-03	2.70E-02	2.87E-03	6.40E-05	1.92E-03	1.06E-02	4.84E-02	3.30E-02
Activity	6.47E-08	3.65E-05	1.19E-06	1.79E-07	4.88E-05	1.24E-07	7.41E-06	2.49E-09
Voice	8.81E-03	2.39E-03	3.45E-03	2.01E-02	7.35E-03	1.86E-04	8.08E-03	1.67E-03
Facial Expression	**4.15E-01**	2.59E-05	1.04E-03	2.12E-07	3.30E-04	1.14E-04	2.71E-04	6.89E-03
Seizure	2.94E-11	7.60E-11	1.18E-10	1.21E-09	9.69E-04	2.97E-11	2.95E-11	3.98E-11
ALL	2.22E-03	**5.08E-01**	1.65E-02	1.03E-02	1.00E-03	3.80E-04	1.52E-02	2.86E-01
Heart	6.52E-07	8.33E-07	5.36E-05	1.30E-09	5.29E-06	1.30E-09	1.32E-07	1.09E-08
Ionosphere	2.53E-04	3.50E-02	**1.00E-01**	7.89E-06	3.26E-04	6.06E-04	5.16E-04	4.17E-06
Wdbc	1.33E-02	**3.68E-01**	1.84E-05	5.02E-03	1.93E-02	5.09E-09	1.22E-04	1.05E-02
Data sets	BPSO		MBPSO	GPSO	MPSOELM	MFOPSO	BBPSOVA	ALPSO
Crohn	1.41E-09		1.23E-05	3.38E-10	6.63E-09	1.64E-05	1.36E-06	1.35E-03
Myeloma	4.06E-10		3.70E-09	1.76E-08	1.21E-09	1.09E-06	3.52E-06	3.78E-09
Arcene	7.05E-07		1.51E-07	1.40E-07	3.06E-07	6.70E-04	4.40E-05	4.58E-07
MicroMass	2.43E-09		9.54E-08	1.13E-05	4.80E-07	9.00E-06	3.26E-04	9.12E-05
Parkinsons	2.97E-02		1.20E-02	5.39E-03	5.00E-05	1.90E-05	2.32E-04	5.81E-03
Activity	6.21E-10		8.34E-10	1.42E-07	4.88E-09	3.97E-08	2.17E-07	6.88E-08
Voice	8.18E-03		7.50E-03	**3.97E-01**	1.16E-02	**1.47E-01**	**2.22E-01**	5.33E-03
Facial Expression	**6.16E-02**		**4.91E-01**	4.89E-02	**3.94E-01**	4.64E-02	2.83E-02	3.61E-02
Seizure	2.93E-11		2.92E-11	3.04E-11	2.90E-11	2.96E-11	2.95E-11	2.97E-11
ALL	5.91E-04		1.09E-02	5.54E-02	5.70E-03	6.72E-03	1.44E-02	2.55E-02
Heart	5.54E-09		1.76E-08	1.96E-08	1.02E-07	1.10E-04	1.18E-06	1.35E-05
Ionosphere	2.88E-02		5.43E-05	1.26E-04	4.39E-04	2.69E-04	1.66E-03	3.35E-04
Wdbc	4.84E-03		2.25E-03	8.69E-03	1.14E-02	2.00E-02	**1.10E-01**	1.07E-02

**Table 8:** The mean results of the number of selected features over 30 runs.

Data Sets	PSO	DE	SCA	DA	GWO	CSO	HPSO-SSM	Catfish-BPSO	Prop. PSOVA1	Prop. PSOVA2
Crohn	9468.8	8942.4	7594.7	8423.4	6292.6	**1151.5**	8846.2	9364.5	7026.6	7697.6
Myeloma	5654.6	5130.2	4462.9	4740.1	3680.3	**1633.5**	5236.9	5476.4	4059.0	4264.5
Arcene	3976.1	4046.1	3388.6	3695.4	2770.4	**2545.3**	3967.2	4424.8	3395.0	3412.4
MicroMass	548.6	527.2	439.8	485.9	**330.6**	1123.0	554.3	588.8	461.3	476.8
Parkinsons	323.3	310.2	266.3	283.2	**209.8**	492.0	323.6	361.6	273.1	274.8
Activity	237.6	222.9	184.0	208.2	**146.3**	394.4	232.7	255.7	194.0	185.5
Voice	128.0	121.4	108.3	118.1	86.7	**65.0**	122.0	140.2	108.6	109.5
Facial Expression	131.4	112.8	88.4	72.0	80.7	**60.1**	84.6	121.6	92.7	97.7
Seizure	61.0	38.4	25.3	33.4	19.7	**5.1**	58.0	39.7	19.4	12.2
ALL	26.5	23.0	18.4	29.5	12.8	**9.5**	25.4	28.8	19.0	15.8
Heart	28.8	23.9	20.9	27.8	**17.8**	56.7	26.4	31.9	21.8	24.6
Ionosphere	12.5	9.3	9.6	11.8	9.4	9.6	11.3	13.1	10.3	**6.9**
Wdbc	9.9	5.5	3.9	9.4	4.73	**3.4**	4.7	10.4	9.8	7.9
Data sets	BPSO		MBPSO	GPSO	MPSO-ELM	MFO-PSO	BBPSO-VA	ALPSO	Prop. PSOVA1	Prop. PSOVA2
Crohn	11,134.8		11,106.7	10,030.2	10,188.4	6886.1	9093.0	9178.7	7026.6	7697.6
Myeloma	6298.8		6299.0	5817.9	5924.5	4073.2	5299.6	5191.3	4059.0	4264.5
Arcene	4977.2		4974.0	4484.6	4541.9	3014.3	4078.2	4051.2	3395.0	3412.4
MicroMass	646.2		641.5	611.5	619.5	439.6	562.1	569.5	461.3	476.8
Parkinsons	378.1		374.4	356.4	360.8	260.2	327.0	310.0	273.1	274.8
Activity	277.2		277.8	261.4	272.9	195.1	237.8	241.7	194.0	185.5
Voice	152.9		148.2	140.0	147.3	101.9	131.1	134.4	108.6	109.5
Facial Expression	146.2		142.0	129.4	135.2	95.7	122.6	115.7	92.7	97.7
Seizure	80.1		74.5	57.2	68.6	38.7	49.9	54.4	19.4	12.2
ALL	35.4		33.3	27.9	33.6	23.1	23.7	31.6	19.0	15.8
Heart	34.0		30.9	32.0	35.0	25.1	27.4	32.0	21.8	24.6
Ionosphere	12.5		10.6	9.1	13.3	8.6	9.1	9.9	10.3	**6.9**
Wdbc	10.8		6.8	9.1	11.8	7.6	8.6	9.6	9.8	7.9

**Table 9:** The mean training computational costs over a set of 30 runs (in seconds).

Data Sets	PSO	DE	SCA	DA	GWO	CSO	HPSO-SSM	Catfish BPSO	Prop. PSOVA1	Prop. PSOVA2
Crohn	3.60E-01	3.16E-01	3.57E-01	3.50E-01	2.96E-01	**2.91E-01**	3.18E-01	3.25E-01	3.47E-01	3.17E-01
Myeloma	3.00E-01	2.78E-01	3.10E-01	2.90E-01	**2.48E-01**	3.00E-01	2.80E-01	2.88E-01	2.90E-01	2.66E-01
Seizure	1.24E+01	1.24E+01	1.25E+01	1.25E+01	1.25E+01	1.25E+01	1.33E+01	1.24E+01	1.26E+01	**1.16E+01**
Data sets	BPSO		MBPSO	GPSO	MPSO-ELM	MFO-PSO	BBPSO VA	ALPSO	Prop. PSOVA1	Prop. PSOVA2
Crohn	3.83E-01		3.55E-01	5.38E-01	3.80E-01	4.90E-01	4.30E-01	4.36E-01	3.47E-01	3.17E-01
Myeloma	3.17E-01		3.09E-01	3.98E-01	3.20E-01	3.59E-01	3.47E-01	3.57E-01	2.90E-01	2.66E-01
Seizure	1.26E+01		1.24E+01	1.27E+01	1.24E+01	1.26E+01	1.25E+01	1.25E+01	1.26E+01	**1.16E+01**

**Table 10:** The mean classification accuracy rates over 30 runs for the mechanisms in PSOVA1 and PSOVA2 using the Seizure and Voice data sets.

PSOVA1	Mean Classification Accuracy Rate		PSOVA2	Mean Classification Accuracy Rate	
	Seizure	Voice		Seizure	Voice
PSO	0.8459	0.8237	PSO	0.8459	0.8237
PSO + Leader enhancement	0.8475	0.8281	PSO + Leader enhancement	0.8463	0.8254
PSO + Leader & worse solution enhancement	0.8510	0.8316	PSO + Leader & worse solution enhancement	0.8495	0.8298
Leader & worse solution enhancement + ameliorated signals	0.8672	0.8491	Leader & worse solution enhancement + exemplar breeding	0.8733	0.8535
Leader & worse solution enhancement + ameliorated signals + spiral search	0.8698	0.8526	Leader & worse solution enhancement + exemplar breeding + coefficient generation	0.8860	0.8632

**Table 11:** Evaluation results for 11 benchmark functions with dimension = 30.

		PSOVA1						PSOVA2			
		Standard PSO	PSO+ Mirroring	1 (Leader Enhancement)	1 + 2 (Worse Enhancement)	1+2+3 (Diverse Signals)	1+2+3+4 (Spiral)	1 (Leader Enhancement)	1 + 2 (Worse Enhancement)	1+2+3 (Exemplar)	1+2+3+4 (Coefficient)
**Ackley**	MEAN	1.97E+01	1.76E+01	1.62E+01	7.19E+00	3.12E+00	1.69E+00	9.33E+00	6.79E+00	1.37E+00	**9.07E-01**
	MIN	1.89E+01	1.46E+01	5.98E+00	5.15E+00	2.11E+00	4.92E-01	2.50E+00	3.67E+00	2.29E-01	**1.36E-01**
	MAX	1.98E+01	1.87E+01	1.99E+01	9.47E+00	4.48E+00	2.44E+00	1.44E+01	8.77E+00	2.43E+00	**2.11E+00**
	STD	**1.68E-01**	9.73E-01	4.81E+00	1.03E+00	4.77E-01	4.95E-01	3.05E+00	1.14E+00	5.90E-01	6.28E-01
**Dixon**	MEAN	2.22E+05	1.17E+03	7.25E+02	6.81E+01	3.98E+01	9.40E+00	1.12E+02	5.09E+01	1.15E+01	**6.49E+00**
	MIN	1.40E+01	1.58E+02	1.03E+02	4.82E+00	8.32E+00	1.66E+00	2.60E+00	4.67E+00	1.88E+00	**1.39E+00**
	MAX	9.77E+05	2.85E+03	2.91E+03	2.01E+02	1.56E+02	**2.72E+01**	3.40E+02	3.44E+02	5.45E+01	3.46E+01
	STD	2.45E+05	9.10E+02	5.37E+02	4.67E+01	3.29E+01	**5.27E+00**	1.38E+02	6.81E+01	1.17E+01	6.74E+00
**Griewank**	MEAN	1.24E+02	1.52E+01	4.54E+00	9.28E-01	4.11E-01	1.76E-01	3.79E+00	9.86E-01	1.76E-02	**6.28E-03**
	MIN	1.04E+00	3.47E+00	1.03E+00	5.99E-01	2.21E-02	2.09E-02	1.40E-01	2.61E-01	4.41E-03	**2.21E-03**
	MAX	2.71E+02	3.16E+01	1.75E+01	1.15E+00	8.34E-01	5.76E-01	9.10E+01	2.13E+00	4.55E-02	**1.46E-02**
	STD	6.48E+01	6.98E+00	3.70E+00	1.66E-01	2.47E-01	1.28E-01	1.65E+01	4.14E-01	1.07E-02	**3.45E-03**
**Rastrigin**	MEAN	3.24E+02	2.43E+02	2.23E+02	1.14E+02	8.54E+01	**5.79E+01**	1.30E+02	1.07E+02	7.71E+01	6.43E+01
	MIN	2.69E+02	1.85E+02	1.48E+02	**2.28E+01**	4.07E+01	2.73E+01	7.59E+01	5.65E+01	4.13E+01	3.45E+01
	MAX	3.96E+02	3.09E+02	3.08E+02	2.28E+02	1.31E+02	9.66E+01	1.78E+02	1.70E+02	1.14E+02	**9.44E+01**
	STD	3.64E+01	2.94E+01	4.05E+01	4.59E+01	2.17E+01	1.84E+01	2.63E+01	2.79E+01	1.86E+01	**1.47E+01**
**Rothyp**	MEAN	1.02E+05	4.39E+04	1.63E+04	1.04E+04	7.69E+02	5.94E+00	1.30E+04	4.41E+03	5.47E+00	**2.11E+00**
	MIN	1.70E+04	2.12E+04	4.23E+03	2.99E+03	2.52E+02	7.97E-01	3.15E+00	2.00E+01	1.29E+00	**4.93E-01**
	MAX	2.07E+05	8.25E+04	3.35E+04	2.48E+04	1.55E+03	1.98E+01	5.90E+04	2.56E+04	1.86E+01	**4.78E+00**
	STD	6.31E+04	1.51E+04	7.49E+03	4.72E+03	3.21E+02	5.14E+00	1.64E+04	6.36E+03	3.95E+00	**1.08E+00**
**Rosenbrock**	MEAN	6.21E+05	2.63E+04	1.12E+04	9.80E+03	3.43E+02	7.43E+01	2.35E+04	2.94E+03	8.48E+01	**6.56E+01**
	MIN	2.84E+05	9.42E+03	3.54E+03	2.76E+03	1.64E+02	**2.52E+01**	5.20E+01	8.18E+01	3.13E+01	3.10E+01
	MAX	1.47E+06	5.45E+04	3.95E+04	2.03E+04	7.57E+02	**1.58E+02**	8.17E+04	2.52E+04	1.90E+02	2.21E+02
	STD	2.32E+05	1.19E+04	8.35E+03	4.72E+03	1.50E+02	**4.07E+01**	2.90E+04	6.26E+03	4.91E+01	5.24E+01
**Sphere**	MEAN	2.81E+01	1.42E+01	8.48E+00	4.04E+00	3.76E-01	9.16E-02	3.53E+00	8.80E-01	6.10E-02	**4.00E-02**
	MIN	1.15E-02	5.75E+00	3.17E+00	1.96E+00	1.79E-01	2.90E-02	6.78E-03	**3.18E-05**	2.70E-02	2.24E-02
	MAX	7.87E+01	2.87E+01	1.81E+01	7.26E+00	7.48E-01	2.07E-01	2.63E+01	2.62E+01	1.08E-01	**7.52E-02**
	STD	2.47E+01	5.35E+00	3.33E+00	1.45E+00	1.39E-01	5.02E-02	9.06E+00	4.79E+00	2.26E-02	**1.33E-02**
**Sumpow**	MEAN	7.07E-02	5.68E-02	1.28E-02	4.52E-03	9.81E-05	**1.24E-06**	3.55E-02	5.02E-03	2.87E-05	2.13E-05
	MIN	9.19E-04	1.10E-03	6.47E-04	1.13E-04	2.22E-06	**1.37E-09**	3.54E-03	1.23E-04	2.27E-06	1.21E-06
	MAX	8.16E-01	1.82E-01	6.53E-02	1.36E-02	4.86E-04	**1.50E-05**	1.80E-01	3.68E-02	1.65E-04	7.90E-05
	STD	1.59E-01	4.33E-02	1.67E-02	4.38E-03	9.77E-05	**2.85E-06**	3.53E-02	8.04E-03	2.98E-05	1.78E-05
**Zakharov**	MEAN	6.27E+02	4.11E+02	3.25E+02	1.70E+02	1.01E+02	8.27E+01	2.99E+02	1.56E+02	9.61E+01	**7.39E+01**
	MIN	5.53E+02	3.38E+02	2.03E+02	7.22E+01	5.65E+01	5.07E+01	2.00E+02	6.55E+01	5.58E+01	**4.81E+01**
	MAX	7.63E+02	4.52E+02	4.32E+02	2.90E+02	1.52E+02	1.49E+02	3.84E+02	2.19E+02	1.34E+02	**1.04E+02**
	STD	5.56E+01	2.81E+01	6.42E+01	4.41E+01	2.20E+01	2.07E+01	4.57E+01	4.12E+01	1.81E+01	**1.46E+01**
**Sumsqu**	MEAN	6.82E+02	4.26E+02	2.38E+02	6.48E+01	5.13E+00	2.95E+00	2.02E+02	4.40E+01	3.99E+00	**2.07E+00**
	MIN	7.92E+01	2.26E+02	1.22E+02	2.56E+01	1.65E+00	6.65E-01	**1.09E-02**	4.40E-02	1.16E+00	6.20E-01
	MAX	1.34E+03	7.25E+02	3.63E+02	1.45E+02	1.04E+01	**8.44E+00**	4.98E+02	3.47E+02	1.12E+01	9.05E+00
	STD	3.35E+02	1.15E+02	7.35E+01	2.83E+01	2.28E+00	1.86E+00	1.39E+02	7.25E+01	2.90E+00	**1.67E+00**
**Powell**	MEAN	4.91E+03	2.85E+03	5.03E+02	4.43E+02	3.72E+01	1.91E+01	3.08E+02	2.34E+02	2.63E+01	**1.02E+01**
	MIN	5.46E+02	4.94E+02	4.01E+02	3.20E+02	1.89E+00	9.97E-01	**2.87E-01**	1.47E+00	7.82E+00	1.83E+00
	MAX	8.11E+03	6.87E+03	6.19E+02	5.62E+02	1.34E+02	9.65E+01	2.96E+03	1.88E+03	9.61E+01	**3.90E+01**
	STD	2.21E+03	1.96E+03	5.86E+01	6.24E+01	2.74E+01	1.89E+01	6.18E+02	4.20E+02	1.83E+01	**7.72E+00**

## Data Availability

The data sets employed in this study are publicly available at the site of UCI Machine Learning Repository, https://archive.ics.uci.edu/ml/datasets.php.
